# Using the concordance of *in vitro* and *in vivo* data to evaluate extrapolation assumptions

**DOI:** 10.1371/journal.pone.0217564

**Published:** 2019-05-28

**Authors:** Gregory S. Honda, Robert G. Pearce, Ly L. Pham, R. W. Setzer, Barbara A. Wetmore, Nisha S. Sipes, Jon Gilbert, Briana Franz, Russell S. Thomas, John F. Wambaugh

**Affiliations:** 1 National Center for Computational Toxicology, U.S. EPA, Research Triangle Park, North Carolina, United States of America; 2 Oak Ridge Institute for Science and Education, Oak Ridge, Tennessee, United States of America; 3 National Exposure Research Laboratory, U.S. EPA, Research Triangle Park, North Carolina, United States of America; 4 Division of the National Toxicology Program, NIEHS, Research Triangle Park, North Carolina, United States of America; 5 Cyprotex, Watertown, MA, United States of America; University of Virginia, UNITED STATES

## Abstract

Linking *in vitro* bioactivity and *in vivo* toxicity on a dose basis enables the use of high-throughput *in vitro* assays as an alternative to traditional animal studies. In this study, we evaluated assumptions in the use of a high-throughput, physiologically based toxicokinetic (PBTK) model to relate *in vitro* bioactivity and rat *in vivo* toxicity data. The fraction unbound in plasma (*f*_*up*_) and intrinsic hepatic clearance (*Cl*_*int*_) were measured for rats (for 67 and 77 chemicals, respectively), combined with *f*_*up*_ and *Cl*_*int*_ literature data for 97 chemicals, and incorporated in the PBTK model. Of these chemicals, 84 had corresponding *in vitro* ToxCast bioactivity data and *in vivo* toxicity data. For each possible comparison of *in vitro* and *in vivo* endpoint, the concordance between the *in vivo* and *in vitro* data was evaluated by a regression analysis. For a base set of assumptions, the PBTK results were more frequently better associated than either the results from a “random” model parameterization or direct comparison of the “untransformed” values of AC_50_ and dose (performed best in 51%, 28%, and 21% of cases, respectively). We also investigated several assumptions in the application of PBTK for IVIVE, including clearance and internal dose selection. One of the better assumptions sets–restrictive clearance and comparing free *in vivo* venous plasma concentration with free *in vitro* concentration–outperformed the random and untransformed results in 71% of the *in vitro-in vivo* endpoint comparisons. These results demonstrate that applying PBTK improves our ability to observe the association between *in vitro* bioactivity and *in vivo* toxicity data in general. This suggests that potency values from *in vitro* screening should be transformed using *in vitro-in vivo* extrapolation (IVIVE) to build potentially better machine learning and other statistical models for predicting *in vivo* toxicity in humans.

## Introduction

Relatively few chemicals in commercial use have been fully evaluated for hazard, in part due to the resource intensive nature of *in vivo* animal testing [1–4]. To address concerns over the potential health effects of data-poor chemicals, new approach methodologies for chemical toxicity testing based on high-throughput *in vitro* and computational tools are being developed by researchers from government, industry, and academia [5]. High-throughput screening assays, such as those used in the Tox21 and ToxCast programs, provide *in vitro* bioactivity data that may inform the potential hazard of a chemical [6, 7]. To link *in vitro* assays with particular *in vivo* endpoints, statistical and machine learning models have been developed that select and weigh the potency and hit call data from relevant assays [8–10]. Using toxicokinetics (TK) may potentially improve performance of such models and elucidate the general correlation between *in vitro* bioactivity and *in vivo* toxicity data [11–13]. Since the probability of a biochemical interaction is proportional to the chemical concentration of ligand at the receptor [14, 15], the 2007 National Academies of Sciences, Engineering, and Mathematics report “Toxicity Testing in the 21^st^ Century” proposed that dose-response modeling using physiologically-based TK (PBTK) models is needed to use high-throughput screening data to estimate chemical risk [16]. TK describes the mathematical relationship between external dose and internal concentrations, accounting for processes including absorption, distribution, metabolism, and excretion of a chemical [13]. Utilizing TK, an *in vitro* bioactive concentration that is suggestive of potential hazard can be extrapolated to an administered equivalent dose (AED) on a mg/kg body mass/day basis, allowing for subsequent comparison to estimated exposure rates [2, 5, 17–21].

Although TK is important for transforming *in vitro* bioactive concentrations to estimate *in vivo* doses, a large majority of chemicals are without TK data [19, 20], including hundreds of chemicals detected in many U.S. citizens by the Centers for Disease Control and Prevention National Health and Nutrition Examination survey [22].To overcome this limitation, high-throughput methods have been developed that use *in vitro* approaches to determine important TK parameters such as plasma protein binding and first order hepatic clearance. *In vitro* TK parameters can then be scaled to *in vivo* situations in a process referred to as TK *in vitro-*to-*in vivo* extrapolation (IVIVE) [21, 23–26]. More broadly, IVIVE methods are critical in the interpretation of *in vitro* toxicity results and enable those results to be understood in the context of risk posed to human health [17, 19, 20]. Wetmore *et al*. 2013 evaluated the effect of including a TK model on the ability of a statistical classification analysis to predict *in vivo* results from *in vitro* toxicity data for the rat [27]. The authors measured *in vitro* rat intrinsic hepatic clearance (*Cl*_*int*_) and fraction unbound in plasma (*f*_*up*_) for 59 chemicals and used in a TK model for IVIVE. A classical TK model was used to convert ToxCast assay AC_50_ (the concentration at 50% of maximum activity) values to AED for comparison to the lowest dose in which any effect was observed from traditional *in vivo* toxicity studies. In general, the *in vitro* assay with the lowest AED was less than the corresponding lowest dose in which any effect was observed for a given compound, demonstrating the potential of TK in estimating hazard. However, the performance of a statistical classification analysis was only slightly improved by incorporating TK. The authors suggested that this could have been due to assumptions in the application of the TK, the interpretation of the *in vitro* assay results, variability in the *in vivo* results, or other factors. Regarding the TK, the authors noted that the use of steady state values, assumption of restrictive clearance (i.e., hepatic clearance dependent on *f*_*up*_ [28]), inability to characterize active renal transport, inaccurate bioavailability assumptions, and extrahepatic metabolism routes may have influenced results. Additionally, assuming a nominal AC_50_ concentration to be bioactive ignores the disposition of the chemical to *in vitro* assay components (e.g., cells, media, aqueous, plastic walls) [11, 29].

One critical assumption in the PBTK model is whether hepatic clearance is dependent or independent of *f*_*up*_ (i.e., restrictive or nonrestrictive) [19]. Restrictive clearance assumes that bound chemical is not made readily available for metabolism and imparts a delay on chemical clearance rates. In contrast, non-restrictive clearance assumes that the off-rates for the plasma protein-bound parent chemicals are sufficiently rapid to instantly replenish the pool of unbound parent in the plasma as needed. Regarding selection of predicted internal concentrations derived from *in vivo* bioassay doses, assumptions are made about which concentration determined from the PBTK model (plasma vs. tissue, mean vs. maximum, total vs. free) would be most comparable to the AC_50_ [11]. Assumptions must also be made regarding the chemical concentration (nominal vs. free) that is bioactive in the *in vitro* assay, and whether additional calculations for distribution of the chemical in the assay are necessary [11]. Rather than use steady-state solutions from a classical 3-compartment model [27], a time-dependent, PBTK model allows for comparison of concentration from a tissue compartment corresponding to the cell type or target organ of an assay [11, 13].

In this current work, the ability of a high-throughput PBTK model to elucidate the general association between *in vitro* bioactivity and *in vivo* toxicity data is evaluated. Performance of the application of the PBTK model in this respect is determined relative to results from a randomization test (i.e., random parameterization of the model) and direct comparison of the untransformed values of dose and AC_50_. Additionally, the influence of some of the main assumptions in the application of the high-throughput PBTK model are investigated. New TK parameters of rat-based *in vitro* measured *f*_*up*_ and *Cl*_*int*_ are reported and were incorporated in the PBTK modeling. Evaluations were carried out for 84 chemicals having TK parameter data and corresponding *in vitro* bioactivity and *in vivo* toxicity data. The *in vitro* bioactivity data used were from 242 ToxCast assay endpoints having observed bioactivities for any of the corresponding chemicals. These were compared to *in vivo* data for rat in two separate analyses: 1) the “endpoint level” analysis used doses at which 68 specific pathologies were observed in ToxRefDB for different study types while 2) the “point of departure” (POD) level analysis used lowest observed effect level (LOEL) and lowest observed adverse effect level (LOAEL) values reported on the EPA’s CompTox dashboard [30]. Regarding the latter dataset, the definition of POD in this work is meant for research purposes and suggests doses that might be benchmarks for the minimum effect in repeat dose toxicity studies.

To thoroughly evaluate the application of PBTK for IVIVE, the model was used for both forward and reverse dosimetry. In forward dosimetry, *in vivo* endpoint level doses for specific pathologies in rat and study level POD were converted to internal concentrations (e.g., mean plasma concentration) for comparison with *in vitro* AC_50_ values. In reverse dosimetry [13, 31], AC_50_ values were converted to AED values [17, 32] for comparison with doses from the endpoint level and POD level data. While one might expect that the TK that works in the reverse application should perform similarly for the forward solution, the comparison of dose vs. AED (reverse dosimetry) is not equivalent to the comparison of AC_50_ and predicted concentration (forward dosimetry) because the transformation between dose and concentration varies across chemicals and *in vivo* study treatment regimens (e.g., the number of doses). The performances of the reverse and forward solutions were evaluated by a series of orthogonal regressions of the standardized log_10_ transforms of the variables. In general, applying the PBTK model elucidates the association between *in vitro* bioactivity and *in vivo* endpoint level toxicity data. Several assumption sets appeared to perform well, including the assumption set of restrictive clearance with the selection of *in vivo* mean free concentration in venous plasma and the *in vitro* free concentration predicted by an *in vitro* disposition model. Additionally, this latter assumption set demonstrated less bias with respect to input model parameters (e.g., *f*_*up*_ and *Cl*_*int*_) than the biases exhibited by otherwise similarly performing assumptions sets.

## Methods

In this work, the parameters *f*_*up*_ and *Cl*_*int*_ were measured *in vitro* for rat and incorporated in a PBTK model. These data were combined with previously published rat-specific *in vitro* TK data collected in the R package *httk* [23]. A PBTK model was used to evaluate two dosimetry approaches for comparing high throughput screening *in vitro* bioactivity and rat *in vivo* toxicity data. A reverse dosimetry approach transformed *in vitro* concentrations to predicted administered equivalent doses. Conversely, a forward dosimetry approach transformed *in vivo* doses to predicted plasma concentrations. We restricted our evaluation to chemicals for which effects were observed for both *in vitro* bioactivity and *in vivo* toxicity data. For each combination of *in vitro* bioactivity and *in vivo* endpoint a regression analysis [24, 33, 34] was used to evaluate the performance of the PBTK model relative to the untransformed values and randomized PBTK results. Results were summarized based on the count of the number of times the PBTK model performed better than both a randomized result (y-randomized TK parameters) and comparison of the untransformed values (*in vitro* AC_50_ vs. *in vivo* dose). Processed data and models are provided in the R package *httk* [23] version 1.9 (https://cran.r-project.org/web/packages/httk/). All analyses were performed in R version 3.5.1. Input data and scripts for analysis are available in S1 File. A list of the abbreviations used in this work is included in S2 File.

### New *in vitro* measured TK parameters

New *In vitro* TK parameters for rat of *f*_*up*_ and *Cl*_*int*_ measured in this work are reported in S1 Table; 67 chemicals had measurable values for *f*_*up*_ and 77 chemicals had measurable values for *Cl*_*int*_. Collectively, 65 chemicals had measured values for both *f*_*up*_ and *Cl*_*int*_. In general, experimental procedures similar to those previously described in [12, 17, 19, 20, 27] were employed for this work.

### Chemical samples

Neat chemicals along with supporting certificates of analysis were obtained from commercial sources by the ToxCast chemical library management contractor (Evotec, San Francisco, CA). Test substances were provided to Cyprotex (Watertown, MA) by Evotec in vials as solids. The solids were dissolved in Dimethyl sulfoxide (DMSO) at 50 mM stock concentration and further diluted as necessary. During chemical concentration analytical method development attempts were made to identify the presence of the chemical peak and to assess the presence of any background peaks. Only chemicals with verifiable presence and minimal background were analyzed for protein binding and/or metabolic stability.

### Chemical analysis

The *in vitro* methods for both *f*_*up*_ and *Cl*_*int*_ require the development of chemical-specific analysis methods to determine relative concentration. This requirement is in contrast to many assays used for high throughput screening in which a single reporter, such as bioluminescence, allows rapid analysis of the degree of perturbation across many chemicals [35]. The methods used here did not develop a calibration curve to quantitate the chemical but rather relied on a percent remaining approach (ratio of chemical peak areas).

For LC-MS/MS the signal is optimized for each compound by electrospray ionization positive or negative ionization mode. A full mass scan is used to optimize the fragmentation voltage and precursor ion m/z. A product ion analysis is used to identify the best fragment for analysis and the optimal collision energy. A test injection is then performed using a standard C18 and/or HILIC column with a water/acetonitrile plus 0.1% formic acid gradient. Samples are analyzed by LC-MS/MS using an Agilent 6410 or AB Sciex 5500 mass spectrometer coupled with an Agilent HPLC and a CTC PAL chilled autosampler, all controlled by MassHunter software (Agilent) or Analyst (AB Sciex). Instrumentation details and parameters for the chemical analysis are also provided in S2 Table and S3 Table.

For some chemicals, peaks could be identified in the hepatocyte incubation media, but could not be separated from the background caused by the presence of plasma protein in the assay for *f*_*up*_ or *vice versa*. This was possibly due to matrix effects (e.g., differential recovery from the plasma matrix compared to the hepatocyte media matrix) that impacted sensitivities in detecting chemicals. As such, *f*_*up*_ and *Cl*_*int*_ were not necessarily determinable for the same chemical.

### Plasma protein binding data analysis (*f*_*up*_)

The fraction of the chemical unbound in the presence of plasma protein (*f*_*up*_) for rats was measured using rapid equilibrium dialysis (RED) [36] in which the free fraction of the chemical was calculated based on the measured chemical concentrations in two chambers separated by a dialysis membrane [17, 19, 27, 36]. Whenever *f*_*up*_ was below the limit of detection, a default value of 0.005 was assumed. This default value was estimated from half the minimum detectable level and previous experience with the RED assay [17]. New measured values for *f*_*up*_ for 67 chemicals are included in the *httk* R package version 1.9 and are available in S1 Table. For use in the models, a correction for non-specific binding in the RED assay was applied following previous methods [24].

Positive controls used in the RED assay were Warfarin (Sigma, Part A2250) and (±)-propranolol hydrochloride (Sigma, Part P0884). DMSO was obtained from Fisher Scientific (Part D128). Assay preparation used acetonitrile (Fisher Scientific, Part A955), water (Fisher Scientific, Part W6), methanol (Fisher Scientific, Part A45, Potassium phosphate buffer pH7.4 (Corning, Part 451201), hydrochloric acid 1N (HCl, Fisher Scientific, Part SA48), and sodium hydroxide (NaOH, Fisher Scientific, Part SS266). Rat plasma was purchased from BioIVT (formerly Bioreclamation; Westbury, NY). Approval by an Institutional Animal Care and Use Committee or equivalent animal ethics committee was not needed.

The plasma was thawed at 37°C using a water and pH adjusted to pH 7.4 ± 0.1 using a concentrated stock solution of either NaOH or HCl. This mixture was dialyzed in a RED Device (Pierce) per the manufacturers’ instructions against phosphate-buffered solution (PBS) and incubated on an orbital shaker. The test compound solutions were diluted into plasma, 5 μM final concentration, where the DMSO concentration did not exceed 1%. The assay was initiated with the addition of 500 μL PBS containing 1% DMSO in the white chamber and 300 μL of the chemical-spiked plasma in the red chamber of the RED device, samples were run in duplicate. The RED device plate was then sealed and incubated in a 37°C incubator with gentle rotating shaking for 4 hours.

The recovery determination plate, or T_0_, was immediately prepared after the start of the 4-hour incubation. For recovery determination, 20 μl of the initial non-incubated plasma was transferred to a 96-well plate and mixed with 50 μl blank PBS containing 1% DMSO followed by the addition of 150 μL cold methanol containing internal standard (1 μM bucetin and 1 μM diclofenac). Blanks were prepared for background subtraction and run in duplicate and prepared by quenching 20 μL plasma containing 1% DMSO and 50 μL PBS containing 1% DMSO into 150 μL internal standard. The quench plate was kept on ice for 10 minutes then centrifuged at ~6000x relative centrifugal force at 4°C for 20 minutes.

After the 4-hour incubation, each well was mixed and 200 μL aliquoted to a transfer plate. Aliquots from both plasma and PBS sides are collected, an equal amount of PBS is added to the plasma sample, and an equal volume of plasma is added to the PBS sample. Methanol containing internal standard is added to precipitate the proteins and release the test agents. From the transfer plate 20 μl of each plasma sample and 50 μl of each PBS sample were aliquoted into a 96-well quench plate. The samples were matrix matched (i.e. 50 μL PBS containing 1% DMSO were mixed with plasma samples and 20 μl plasma containing 1% DMSO were mixed with PBS samples). The samples in the quench plate all received 150 μL of cold methanol containing internal standard. The quench plate was kept on ice for 10 minutes then centrifuged at ~6000x relative centrifugal force at 4°C for 20 minutes. The supernatant was then transferred to a liquid chromatography tandem mass spectrometry (LC-MS/MS) plate or gas chromatography-mass spectrometry (GC-MS) vial for analysis. Each assay was run with propranolol and warfarin as controls.

### Intrinsic hepatic clearance (*Cl*_*int*_) assay

The *in vitro* intrinsic hepatic clearance (i.e., rate of first order metabolic clearance of the parent compound normalized to cell number) in primary rat hepatocytes was measured and analyzed similar to previously described methods [19, 27] at concentrations of 1 μM and 10 μM for each chemical. If the chemical was measurable but disappearance of parent compound was not apparent during the two hours over which the assay was performed, then the clearance was assumed to be zero. For the subsequent calculations the clearance at 1 μM was used, if measured successfully, otherwise the clearance at 10 μM was used. New measured values of rat *Cl*_*int*_ for 77 chemicals are reported in the *httk* R package version 1.9 and are available in S1 Table. *In vivo* clearance is estimated from the *in vitro* measured *Cl*_*int*_ following methods in Wambaugh et al. [26], including division by the estimated unbound fraction in the *in vitro* clearance assay [37], correction using a well-stirred model [38], and scaling by the number of cells in the liver.

For the *Cl*_int_ assay, reference chemicals used were (±)-verapamil hydrochloride (Sigma, Part V4629) and Warfarin. Pooled male cryopreserved rat hepatocytes were purchased from BioIVT. Characterization of hepatocytes, including enzyme expression levels, was performed by the vendor (BioIVT). 500 mL of William’s E medium, 5 mL of 200 mM L Glutamine (final concentration 2 mM) and 3 g of HEPES (final concentration 25 mM) were added to the hepatocytes. The media was mixed by inversion, warmed to 37°C, and adjusted to pH 7.4 before each use. The cells were thawed, viable cells counted, and then equilibrated according to the supplier’s directions. Pooled male cryopreserved rat hepatocytes were added to an incubation plate and kept at 37°C in the incubator. After 30 min equilibration with gentle agitation, 250 μL of test compound solutions were aliquoted in triplicate into wells containing the cells to give the desired final concentration of 1 or 10 μM. The cell suspension was incubated at 37°C as above. At 15, 30, 60 and 120 minutes, 100 μL aliquots from the incubation plate were removed and precipitated into a quench plate containing an equal volume of cold methanol containing internal standard (1 μM bucetin, 1 μM propranolol, and 1 μM diclofenac). The 0 minute sample was prepared from aliquoting 50 μL hepatocytes into a quench plate containing 100 μL internal standard followed by the addition of 50 μL from the compound plate. Blanks were prepared for background subtraction and run in duplicate and prepared by quenching 50 μL hepatocytes and 50 μL media into 100 μL internal standard. The quench plates were kept on ice for 10 minutes then centrifuged at ~6000x relative centrifugal force at 4°C for 20 minutes. The supernatant was then transferred to a LC-MS/MS plate or GCMS vial for analysis. Each assay was run with verapamil, midazolam, and 7-OH 4-trifluoromethyl coumarin as controls.

### Literature data on *in vitro* TK

The prior version 1.8 of the R package *httk* included literature data on rat-specific measurements of *f*_*up*_ and *Cl*_*int*_ for 97 chemicals. For *f*_*up*_ these data were composed of 58 chemicals from Wetmore et al. 2013 [27], 19 chemicals from Wood et al. 2017 [39], 13 chemicals from Pearce et al. 2017 [23], and 7 chemicals from Naritomi et al. 2003 [40]. For *Cl*_*int*_ these data were composed of 59 chemicals from Wetmore et al. 2013 [27], 35 chemicals from Wood et al. 2017 [39], and 3 chemicals from Naritomi et al. 2003 [40].

### Toxicity data

Two sets of *in vivo* data were analyzed in this work: one set for an “endpoint level” analysis and another for a “POD level” analysis. For the analysis of *in vivo* endpoint level data, doses corresponding to observation of specific pathology endpoints were determined for each chemical, and study types were analyzed separately. For the analysis of POD level data, minimum doses were determined for each chemical from combined a dataset of LOEL and LOAEL values determined across studies and study types. While allowing for simultaneous comparisons for a larger number of chemicals, a potential lack of concordance between doses across study types may be expected in the POD analysis. This serves as a point of comparison for the endpoint level analysis. Data for *in vivo* doses used in the endpoint level analysis and the POD level analysis are available in S4 Table and S5 Table, respectively.

### *In vivo* endpoint level data

*In vivo* data for rat were accessed from the Toxicity Reference (ToxRef) database [41–44] version 1. Much of the data in ToxRefDB v1 was derived from studies or study summaries for study designs compliant with or similar to the EPA OCSPP 870 series guidelines [45]. ToxRefDB v1 is a “positives-only” database, and *in vivo* data were reported as the nominal dose at which an effect (not necessarily critical) was observed for a particular endpoint (e.g., nonneoplastic liver change, change in body weight gain, etc.), along with additional information including the chemical used and study descriptors. The analysis in this work included chronic (2 year), subchronic (90 day), and developmental (parental and fetal generations) study types, which were analyzed separately. Multigenerational studies were not evaluated as they were not directly amenable to treatment with the PBTK model as the time units were given in generations. Entries with rat *in vivo* data were selected for analysis. *In vivo* endpoints were defined for each response pathology, as described by each unique combination of study type, effect-category, effect-type, and effect-target. For a given combination of study, chemical, and response endpoint, the lowest dose at which a response was observed was taken as the endpoint level dose. In this respect, the analysis was restricted to positive effects and multiple endpoints from a given study were represented for a given chemical. The doses may therefore differ from LOEL and LOAEL values taken across the study and should not be considered study-level LOEL or LOAELs as they are instead endpoint specific. Also, some chemicals had results from multiple studies. Both sexes and all rat strains were used. The list of *in vivo* endpoints in the analysis are reported in S6 Table.

### *In vivo* POD level data

In contrast to the endpoint specific *in vivo* data, we also evaluated POD level data using LOEL and LOAEL maintained on the CompTox Chemicals dashboard (https://comptox.epa.gov/dashboard). A table of the values used in this analysis (accessed March 2018) is provided in S5 Table. Rat data were selected, entries without usable dose units (mg/kg/day) or duration of study were filtered out, and the study types included in the evaluation were subacute, subchronic, and chronic. Evaluation was further restricted to those doses that were orally administered and had non-zero values. For each chemical, the minimum dose from a combined set of LOEL and LOAEL values across the available data was determined and used in subsequent POD level analyses. Although using data across study type may allow for comparisons made with a larger number of chemicals, data taken across study types and pathologies may have a higher variance.

### *In vitro* bioactivity data

The Toxicity Forecaster (“ToxCast”) project consists of a suite of *in vitro* high throughput screening assays conducted using a variety of different technologies and preparations [46]. ToxCast assays include both biochemical and cell-based assays. These assays are primarily focused on human toxicity and are generally derived from human proteins and tissues. Assays are conducted in concentration-response format and are analyzed to determine if a concentration-dependent response is observed. These responses are modeled by either a Hill function or a Gain-Loss model, which can be summarized by the concentration required for 50% activation (AC_50_). *In vitro* data were accessed from the ToxCast summary files from October 2015 [47]. The AC_50_ value (μM) for each assay component endpoint was used for the evaluations in this study. We restricted our analysis in this work to *in vitro* bioactivity data with positive results (i.e., positive hit-calls) and filtered by disregarding positive hit-calls with any curve-fitting flags. *In vitro* bioactivity data are available in the S1 File.

### Analysis

**Fig 1:**
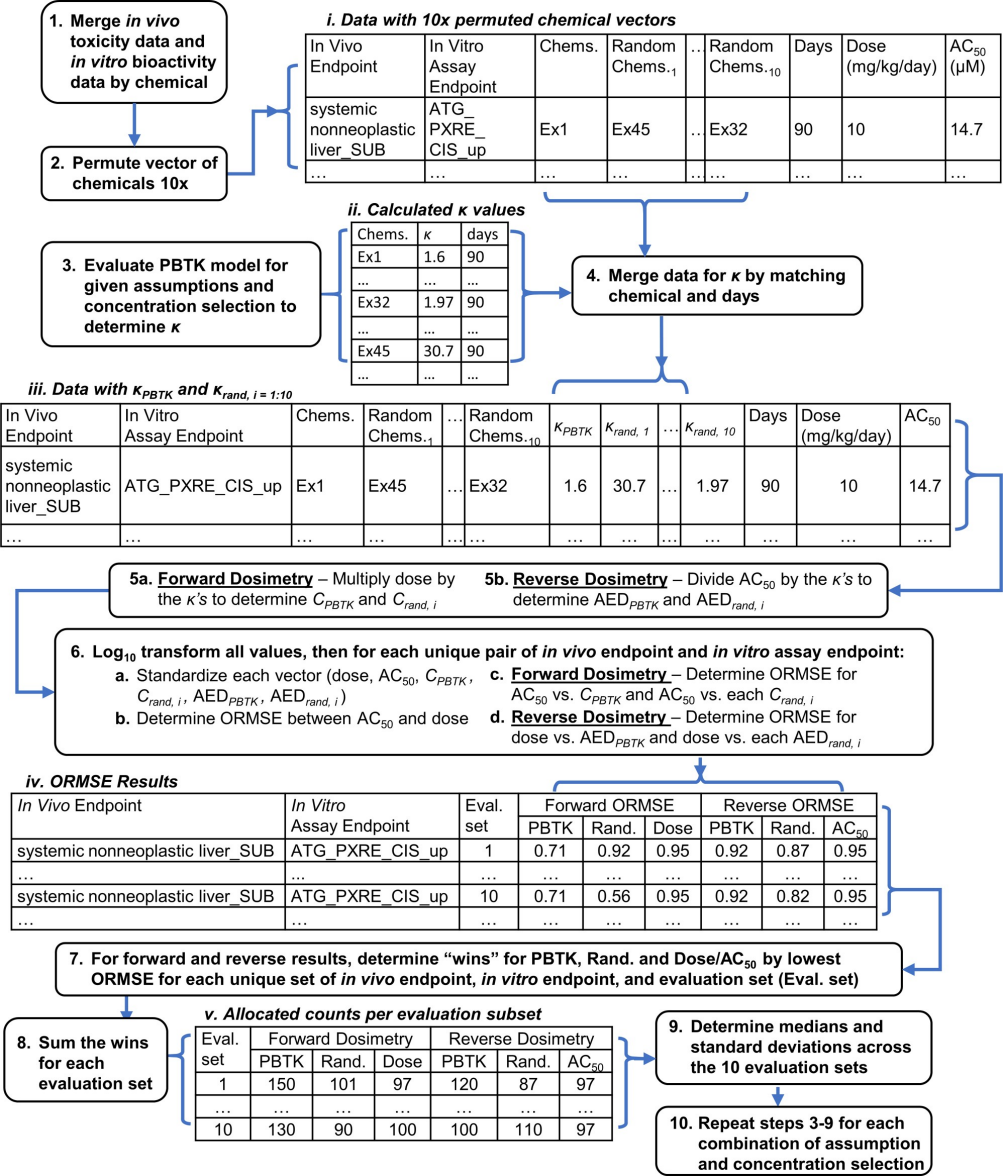
Diagram for the analysis workflow. κ is the ratio of internal concentration (μM) to dose (mg/kg/day) determined using the PBTK model for an external dose of 1 mg/kg/day. ORMSE is the orthogonal root mean square error. Example chemicals are denoted by Ex1, etc. to enable demonstration of data mergers.

An overview of the analysis workflow we used is shown in Fig 1. This workflow applies to both the endpoint level data and POD level data. Scripts to reproduce the analysis are available in the S1 File. As a first step, data for *in vitro* bioactivity and *in vivo* toxicity were merged by chemical. In the endpoint level analysis (for which doses correspond to observed effects for specific pathologies in rat), the number of unique chemicals with values for both dose and AC_50_ were counted for each combination of *in vivo* endpoint and *in vitro* assay. Only those *in vitro*-*in vivo* comparisons with at least 5 chemicals were included in the subsequent analyses. In the POD level analysis (for which doses correspond to minimum values by chemical from combined data for LOEL and LOAEL taken across pathologies and study types), *in vitro* assays in the merged data set having AC_50_ values and corresponding *in vivo* POD doses for at least 5 chemicals were included in subsequent analyses.

### Randomization test

The resultant merged data sets each contained a vector of chemicals having a particular distribution. In addition to comparing the results of IVIVE using the PBTK model relative to untransformed values of dose and AC_50_, we also compared performance of the PBTK model relative to randomized results. If the performance of the random results were similar to the PBTK result, it would suggest that differences between the performance of the PBTK result and untransformed values were due to the mechanics of the model or analysis, rather than chemical specificity. In each of the two separate analyses of *in vivo* data (endpoint and POD level data), the vector of chemicals from the combined data set was permuted ten times with resampling (Fig 1, step 2). Subsequently, these ten vectors of randomly permuted chemicals were used to produce ten sets of “randomized” results. Effectively, each randomized result was determined by parameterizing the PBTK model and accessory calculations based on the corresponding random chemical.

### PBTK model

**Fig 2:**
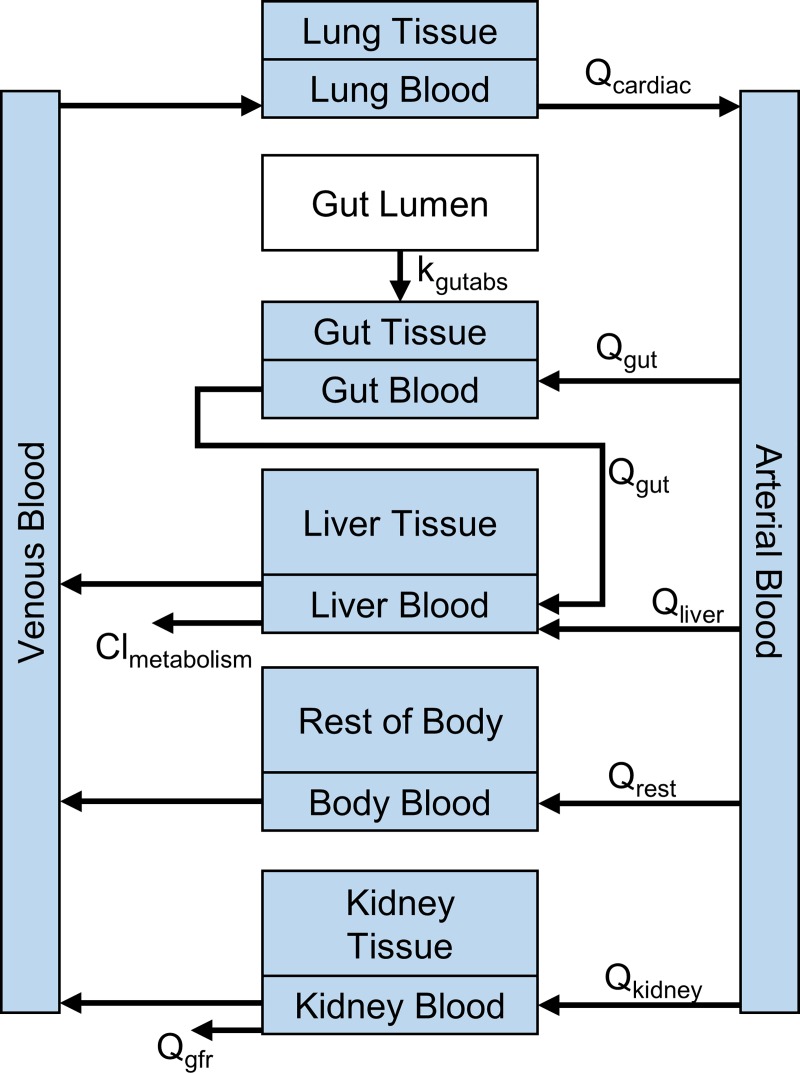
Diagram of the PBTK model in the httk R package. Q represents flow rates, Cl indicates hepatic clearance, k indicates absorption rate [23].

A diagram of the PBTK model used in this work is shown in Fig 2. The model allows for doses to be absorbed through the gut or injected intravenously, although analysis was restricted to oral doses in this work. The fraction absorbed was assumed to be 100%. We accounted for organ and tissue partitioning in the PBTK model following the methods described by Pearce et al. [24]. When experimental values for log P (EPI Suite, [48]) were unavailable, predicted values from the OPERA model [49] were used. pKa values were from the work by Strope, *et al*. [50] or predictions from ChemAxon (Budapest, Hungary). The time course internal concentration of the PBTK model was evaluated using the EPA’s *httk* R package.

For a given set of assumptions, a dose of 1 mg/kg/day administered once a day was used to determine *in vivo* concentration-to-dose ratios (*κ*) by the application of the PBTK model and any accessory calculations with respect to the dose and study length. *κ*, effectively the ratio of the concentration (μ*M*) at 1 mg/kg/day to the dose of 1 mg/kg/day, is determined from the PBTK model and any additional calculations (e.g., concentration selection, *in vitro* disposition). As such, *κ* is a function (generically denoted by *f*) of the chemical, time, assumptions, and internal concentration selection (Eq 1). Concentrations (*C*) produced by the PBTK model are proportional to dose (Eq 2); this holds for concentrations at a given point in time, linear means, maxima, and steady state concentration. This characteristic allows for simple application of reverse dosimetry, where *in vitro* concentrations (i.e., AC_50_) are converted to administered equivalent doses (AED) by dividing the concentration by the corresponding concentration determined for a dose of 1 mg/kg/day (Eq 3) [12, 17, 19–21, 27]. In previous work, this type of conversion was applied for steady state concentrations [20]. The same method is applicable to the transient solution, but with the requirement that the dosing regimen (study length and doses per day) are model inputs [23, 26].

κ=f(1mg/kg/day,chemical,time,assumptions,doseselection) (1)

C=dose*κ (2)

$$AED=AC{}_{50}/\kappa$$ (3)

In the analysis workflow (Fig 1, step 4), values for *κ* determined for different study lengths and chemicals were then merged with the data set by matching chemical and study length to produce the vector of *κ*_*PBTK*_. Similarly, ten separate vectors of random results (*κ*_*rand*,*i* = 1:10_) were produced by assigning *κ* values corresponding to the permuted chemicals and study lengths (Fig 1, table *iii*). It is important to note that only the TK and physicochemical parameters are randomized in this analysis, the initial inputs (dose and study length; AC_50_) remain specific to the original vector of chemicals. Predicted values for AED and internal concentration were then determined following Eqs 2 and 3. Various combinations of the assumptions in the application of the PBTK model for IVIVE were evaluated, each producing a unique vector of *κ*_*PBTK*_ and 10 unique vectors of *κ*_*rand*,*i*_.

### Forward and reverse dosimetry

The models employed in this work are phenomenological in that they are structured to represent the physical processes that have been observed experimentally. As such, if internal concentration (determined from external dose) were associated with AC_50_, then AED (determined from AC_50_) may be expected to be similarly associated to external dose. To evaluate this, we performed two separate comparisons (Fig 1, step 5a-b):

- Forward dosimetry: Evaluate the strength of the association between *in vitro* AC_50_ and predicted *in vivo* concentration (*C*_*PBTK*_) corresponding to dose as determined following Eq 2. The association between *C*_*PBTK*_ and the *in vitro* AC_50_ was compared with the association between *in vitro* AC_50_ and the non-PBTK adjusted external dose values. To evaluate performance relative to chance, the randomized results (*C*_*rand*, *i = 1*:*10*_) were predicted ten times using TK for permuted (random) chemical identifiers and the associations between *in vitro* AC_50_ and each *C*_*rand*, *i = 1*:*10*_ were determined.
- Reverse dosimetry: Evaluate the strength of the association between *in vivo* dose and predicted external dose (AED_*PBTK*_) determined following Eq 3. The association between these doses was compared to the strength of the association between the non-PBTK adjusted *in vitro* AC_50_ and dose. Again, to evaluate performance relative to chance randomized TK result (AED_*rand*, *i = 1*:*10*_) were calculated using the permuted chemical identities.

### Model selection

For each comparison of *in vitro* assay and *in vivo* endpoint, a series of univariate, orthogonal regressions were performed for the standardized log_10_ transforms of the variables (Fig 1, step 6). First, all values (dose, AC_50_, *C*_*PBTK*_, *C*_*rand*, *i*_, AED_PBTK_, AED_rand, i_) were log_10_ transformed. Values were then standardized by subtraction of the mean and division of the standard deviation for the respective variable following Eqs 4 and 5. In this subsection, generic variables (y, x, φ, and θ) are used to describe calculations. For forward dosimetry, the y-variable is log_10_ *in vitro* AC_50_ and the x-variables are log_10_ transforms of *C*_*PBTK*_, *C*_*rand*, *i*_, and *in vivo* external dose. For reverse dosimetry, the y-variable is log_10_ *in vivo* external dose and the x-variables are log_10_ transforms of AED_*PBTK*_, AED_*rand*, *i*_, and *in vitro* AC_50_.

$$\phi_{i}=\frac{y_{i}-\bar{y}}{\sigma_{y}}$$ (4)

$$\theta_{i}=\frac{x_{i}-\bar{x}}{\sigma_{x}}$$ (5)

The statistic used as the basis for the comparisons made in this work is the root mean square error based on the orthogonal distance between a point and the regression line. Both standardized variables are centered on zero and have a variance of 1, subsequently the orthogonal regression line is either φ = θ or φ = -θ. In either case, we can define a root mean square error based on the orthogonal distance between a point and the φ = θ line, which we refer to as the orthogonal root mean square error (ORMSE):

$$ORMSE=\sqrt{\frac{\sum_{i=1}^{n}{\left(\left(\phi_{i}-\theta_{i}\right)/\surd 2\right)}^{2}}{n}}$$ (6)

Effectively, the ORMSE serves as a representation of the association between the two variables (i.e., a lower ORMSE indicates a stronger, more quantifiably definable relationship). For forward dosimetry, ORMSEs were determined between *in vitro* AC_50_ and *C*_*PBTK*_, between AC_50_ and each of the ten sets of *C*_*rand*, *i*_, and between *in vitro* AC_50_ and external dose. For reverse dosimetry, ORMSEs were determined between *in vivo* external dose and AED_*PBTK*_, between external dose and each of the ten sets of AED_*rand*, *i*_, and between external dose and *in vitro* AC_50_. As noted previously, *C*_*rand*, *i*_ and AED_*rand*, *i*_ represent single sets of the ten random results determined by parameterizing the PBTK model and other calculations using random sampling from a distribution of chemicals.

Resulting ORMSE can be organized as in Fig 1, table iv. The ten separate sets of random results allow for ten comparisons to be made for each *in vitro* assay and *in vivo* endpoint comparison. Note that the ORMSE between y = external dose and x = AC_50_ is equivalent to the ORMSE between y = AC_50_ and x = external dose. The only values that change with evaluation subset for a given *in vitro-in vivo* endpoint comparison are the ORMSE for the randomized result. The forward and reverse dosimetry results were treated separately. For a given *in vitro-in vivo* endpoint comparison, ORMSE were compared for each evaluation subset, and “wins” were allocated to the predictor (PBTK, Rand., or Dose/AC_50_) with the lowest ORMSE (Fig 1, step 7). The number of times a predictor had the smallest ORMSE were then counted (Fig 1, step 8) and summarized per evaluation subset (Fig 1, table v). The medians and standard deviations of the counts allocated to each predictor were then determined across the ten evaluation subsets, again treating forward and reverse dosimetry separately (Fig 1, step 9). This process (Fig 1, steps 3–9) was then repeated for every combination of assumption and concentration selection (internal concentration metric and *in vitro* distribution) of interest. An analogous methodology was carried out for the POD level analysis, noting that doses in that case were determined across *in vivo* response types.

### Assumptions evaluated

In addition to understanding the overall impact of applying our high-throughput PBTK model for IVIVE, we also evaluated the influence of some of the main assumptions in the application of the high-throughput PBTK model. These include restrictive vs. nonrestrictive hepatic clearance, selection of concentration from the PBTK model, and application of the Armitage model [51] for distribution of chemicals *in vitro*. A list of the evaluated assumptions is included in S3 File.

### Restrictive vs nonrestrictive hepatic clearance

Hepatic clearance is influenced by the effective rate of desorption of a chemical from the plasma protein to which it is bound [52]. When this rate is relatively slow, the clearance is dependent on the fraction of unbound chemical in the plasma (*f*_*up*_) and therefore considered restrictive. When the desorption rate is fast, the clearance is independent of *f*_*up*_ and termed nonrestrictive. In the PBTK model, restrictive clearance may be accounted for by multiplying the intrinsic hepatic clearance, *Cl*_*int*_, by *f*_*up*_. For limited sets of chemicals with known *in vivo* clearance, assuming nonrestrictive clearance may potentially provide more accurate results, although certain chemicals (e.g., warfarin) are known to be metabolized under restrictive clearance [19, 52].

### Selection of concentration from the PBTK model

The PBTK model produces concentration vs. time profiles for each tissue/compartment in the model. Different concentrations taken at different times may either be more or less indicative of the conditions that lead to an observed *in vivo* response [28] and either more or less relatable to an *in vitro* AC_50_ value. Mean and maximum concentrations were extracted from the solution of the PBTK model for the venous plasma concentration and the tissue plasma concentration from each tissue compartment in the model. The mean concentration is equivalent to the area under the concentration vs. time curve divided by the time-length of the dosing. When comparing predicted *in vivo* concentration (e.g., in plasma) with *in vitro* AC_50_, it may also be preferable to compare concentrations from similar compartments. AC_50_ may either be 1) assumed to be a nominal concentration directly comparable to *C*_*PBTK*_, or 2) assumed to be similar to a free concentration so that *C*_*PBTK*_ should be converted to a free concentration by multiplying *C*_*PBTK*_ by *f*_*up*_. Furthermore, in cases where a model of the distribution of chemical *in vitro* is incorporated in the interpretation of *in vitro* results, it may be that the *in vitro* free concentration should be compared to the predicted *in vivo* unbound concentration, *f*_up_*C_*PBTK*_.

### Distribution in *in vitro* cell-based assays

The distribution of a chemical in an *in vitro* environment results in different concentrations for different assay compartments (cells, water, lipids, proteins). The concentration in a particular assay compartment may be better associated with bioactivity *in vivo* than the nominal concentration (total amount per total well volume). This is particularly true for cell-based assays where there is more material (cells, serum) available for chemical binding. For *in vitro* cell-based assays, we evaluated an updated version of the *in vitro* distribution model by Armitage et al. 2014 [51]. Updates include absorption to the walls, distinction between storage and membrane lipids in the cells, and changes in methods to estimate membrane concentrations. Assay well geometry, media volume, and cell volume were taken from values reported by Corning [53] that correspond to the assay footprint (*i*.*e*., well number). Other descriptors of assay components, including fetal bovine serum (FBS) and cell content (lipid, protein, and water fractions), are taken as averages of those values reported by Armitage. The values for water solubility and Henry’s law constant used were from experimental values in EPI Suite [48] if available or predictions from OPERA [49]. The aqueous phase concentration estimated from the Armitage model was used for subsequent comparisons. For a given *in vitro-in vivo* comparison, corresponding aqueous phase concentrations were calculated for each *in vitro* result. A factor was then defined by the aqueous concentration divided by the nominal AC_50_. The result for including the *in vitro* distribution model was then given by the concentration from the PBTK model divided by the factor, thereby producing a unique *κ*_*PBTK*_ for the given set of assumptions.

## Results

Since the propensity for bioactivity is proportional to chemical concentration, TK frames the dose-response relationship and associated hazard characterization by linking external exposures (theoretical or relevant, depending on the scenario) to resultant internal concentrations. This work evaluates varying sets of IVIVE assumptions by examining the impact of a PBTK model on the association between *in vitro* bioactivity and *in vivo* toxicity data. *In vitro* HTTK parameters of *f*_*up*_ and *Cl*_*int*_ were measured for 65 new chemicals and analyzed jointly with data from the literature for 97 chemicals. For the data considered, TK parameters, *in vitro* AC_50_, and *in vivo* doses were simultaneously available for 84 chemicals. Forward and reverse dosimetry results were determined using the PBTK model for each set of assumptions, and comparisons were made using a regression analysis. Two different *in vivo* data sets were used for the basis of this analysis: the endpoint level data containing 80 chemicals with doses corresponding to observed responses for 106 specific endpoints (68 pathological responses and 3 study types), and the POD level data containing 84 chemicals where effects had been aggregated into a single point of departure per chemical (the minimum LOEL-LOAEL taken across all available pathologies, studies, and study types). For the endpoint level data, different *in vitro* assay–*in vivo* endpoint combinations had different numbers of chemicals that were both active *in vitro* and that had a particular *in vivo* endpoint observed; 2787 endpoint combinations had at least 5 chemicals, while 48 combinations had at least 20. For the comparisons made between the POD level data and *in vitro* assay endpoints, there were 69 comparisons with at least 5 chemicals that were active *in vitro* and *in vivo*, while 17 comparisons had at least 20 chemicals.

For each chemical, time dependent mean and maxima plasma and tissue concentrations were determined by the PBTK model for a dose of 1 mg/kg/day for various study lengths. Model linearity of the ratio of internal concentration to external dose for a given time-point allowed for extrapolation to various dosing scenarios and evaluation of forward and reverse dosimetry. Comparisons were made based on the ORMSE of the standardized log_10_ transforms of the variables; depending on the direction of the dosimetry, AC_50_ (forward) or dose (reverse) was compared vs the PBTK result (internal concentration or AED) relative to a randomized result (internal concentration or AED) and the untransformed value (dose or AC_50_). While other parameters could have been selected, ORMSE was deemed appropriate as a basis for the broad evaluation in this work. Results were compiled by the number of times a variable (PBTK, random, or untransformed) had the lowest ORMSE from each *in vitro* assay and *in vivo* endpoint comparison.

### Example regression

**Fig 3:**
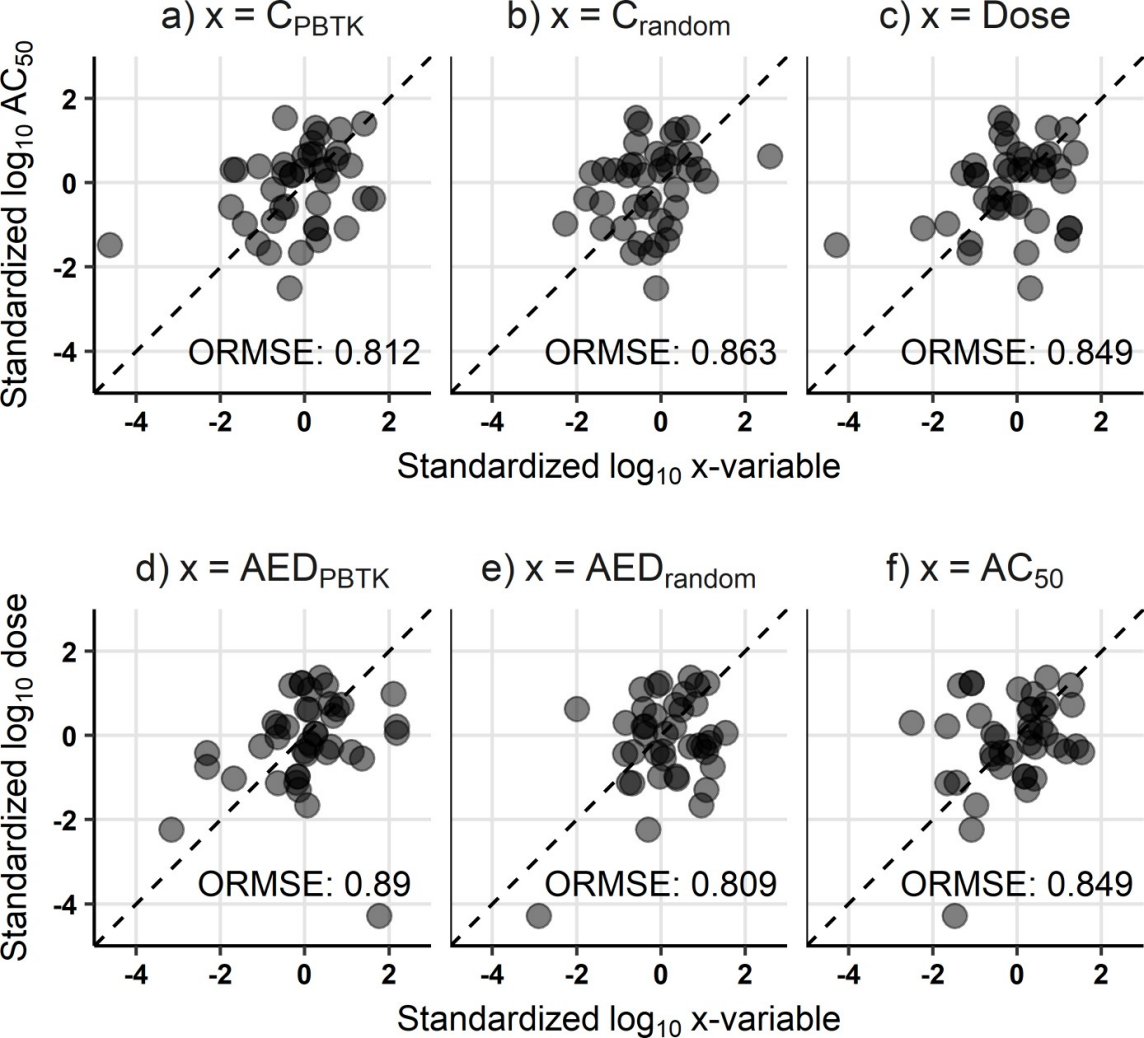
Example regressions of the standardized, log_10_ transforms of variables. Results are shown for forward dosimetry (C_PBTK_, C_random_, Dose; a-c, respectively) and reverse dosimetry (AED_PBTK_, AED_random_, AC_50_; d-f, respectively) from the endpoint level analysis for the *in vitro* assay endpoint of ATG_PXRE_CIS_up and the *in vivo* effect of systemic, nonneoplastic liver pathology from chronic studies. The dashed lines along y = x are the best fit lines for the standardized variables and the corresponding ORMSE (orthogonal root mean square error) are also reported; units are dimensionless.

To illustrate features of the regressions, an example analysis is demonstrated assuming nonrestrictive hepatic clearance in the PBTK model, as well as using the total mean venous plasma concentration and the nominal AC_50_. The results from the Attagene assay endpoint, ATG_PXRE_CIS_up, characterizing the regulation of the pregnane X receptor transcription factor response element in HepG2 cells [54], were compared with the endpoint level *in vivo* data from chronic studies for systemic, non-neoplastic, liver pathology. Outside of this example, each *in vivo* endpoint was compared with every *in vitro* assay endpoint for the complete analysis; more than 750,000 regressions were made in total across all the analyses. For the example case, regressions of the standardized, log_10_ transformed variables are shown in Fig 3 for forward dosimetry (a-c) and reverse dosimetry (d-f). A lower ORMSE indicates a stronger association. The lowest ORMSE in the forward solution comparison is achieved using the PBTK model (Fig 3A). However, for the AED comparison, the lowest ORMSE is for random (Fig 3E). As expected, the ORMSE is the same for AC_50_ vs dose in the forward dosimetry comparison and dose vs AC_50_ in the reverse dosimetry comparison (Fig 3C and 3F); in these latter two comparisons, the points are mirrored around the y = x line, so the orthogonal distance to y = x does not change.

For the PBTK and random results, the difference between the forward dosimetry and reverse dosimetry results is not a simple switching of the axis. Rather, the comparisons of AC_50_ vs concentration are distinct from those for dose vs AED. In Fig 3A (AC_50_ vs C_PBTK_), there is a possible outlier near x = -4 and y = -2 (all variables were standardized and log_10_ transformed, as described in Methods); in the corresponding reverse dosimetry plot (Fig 3D), this is the point at y = -4 and x = 2. If this point were omitted, the trends with respect to ORMSE would be similar for the forward and reverse comparisons. However, this would be a subjective alteration; instead our interest is in evaluating the effect of TK in general across comparisons of a large range of assay endpoints and *in vivo* effects. A broad comparison among various *in vitro* and *in vivo* data should mitigate the influence of any outliers; the example regression shows results for only one of the comparisons that were made, and only one of the ten sets of random results.

**Fig 4:**
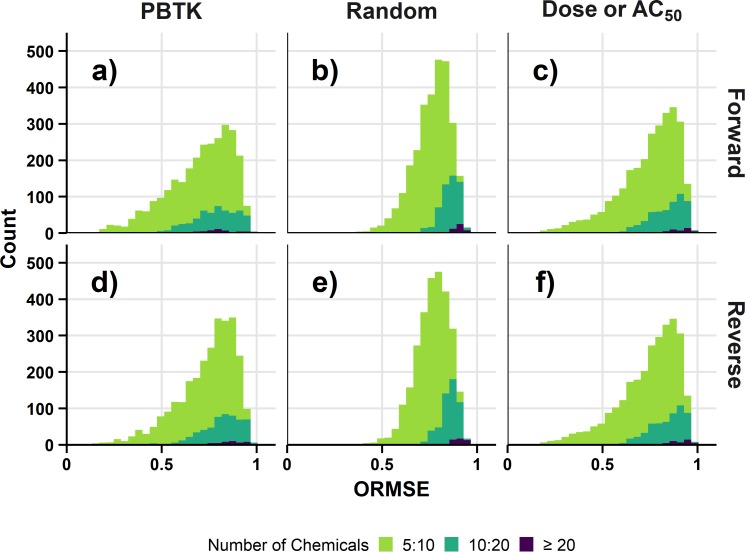
Distributions of ORMSE for the endpoint level analysis. Results are for the assumption set of nonrestrictive clearance and mean total plasma concentration. The number of unique chemicals in the comparison are indicated by fill color. The ORMSE for the random result are median values across the ten sets of results. Figure panels show results for combinations of different ORMSE results and dosimetry: a) PBTK-Forward Dosimetry, b) Random-Forward Dosimetry, c) Dose or AC_50_-Forward Dosimetry, d) PBTK-Reverse Dosimetry, e) Random-Reverse Dosimetry, and f) Dose or AC_50_-Reverse Dosimetry.

**Fig 5:**
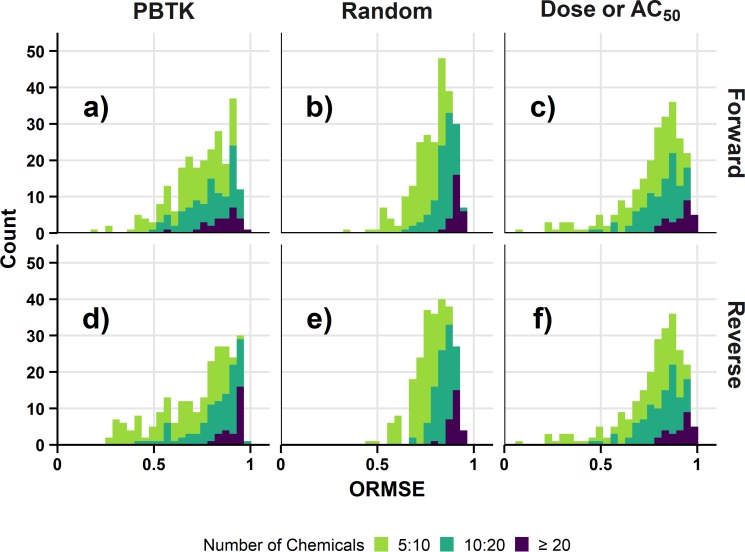
Distributions of ORMSE for the POD level analysis. Results are for the assumption set of nonrestrictive clearance and mean total plasma concentration. The number of unique chemicals in the comparison are indicated by fill color. The ORMSE for the random result are median values across the ten sets of results. Figure panels show results for combinations of different ORMSE results and dosimetry: a) PBTK-Forward Dosimetry, b) Random-Forward Dosimetry, c) Dose or AC_50_-Forward Dosimetry, d) PBTK-Reverse Dosimetry, e) Random-Reverse Dosimetry, and f) Dose or AC_50_-Reverse Dosimetry.

Fig 4 shows the distribution of ORMSE for the endpoint level analysis with the assumptions of nonrestrictive clearance and mean total plasma concentration across all comparisons of *in vitro* assay and *in vivo* endpoint. For demonstrative purposes, the random results in this case are median values from the 10 sets of results. Results are restricted to those comparisons having at least 5 different chemicals. The distributions for the forward and reverse solutions are identical for dose (forward solution comparison) or AC_50_ (reverse solution comparison). Results are also similar between the forward and reverse solutions for the random result, but differences are apparent between forward and reverse dosing for the PBTK result and are particularly noticeable for those comparisons with greater than 20 chemicals. In that case, the reverse dosimetry result (median ORMSE: 0.87) is shifted towards slightly higher values of ORMSE than the forward dosimetry result (median ORMSE: 0.80). Relative to the random result (median ORMSE: 0.91 for forward and reverse, ≥ 20 chemicals), the PBTK result is slightly lower in either case. The PBTK result is also slightly lower than the comparison of untransformed dose and AC_50_ (median ORMSE: 0.90, ≥ 20 chemicals). Similar trends are observed for the corresponding POD level analysis across all the assays in Fig 5. For comparison based on at least 20 chemicals, the reverse result again appears shifted towards higher values of ORMSE (median: 0.94) than the forward solution (median ORMSE: 0.88). The example analysis for the assumption set of nonrestrictive clearance with mean total plasma concentration suggests differences in the results for forward dosimetry and reverse dosimetry. To determine if these differences occur in general, the analysis was extended across various previously described combinations of assumptions and dose metrics.

### Evaluating results across all assumption sets

For the endpoint level data, ORMSE values were determined for the forward and reverse dosimetry comparisons for each *in vitro* assay endpoint and *in vivo* endpoint. For the POD level data, ORMSE were determined for each assay endpoint. The number of times a variable yields the lowest ORMSE were counted for the forward and reverse dosimetry results using both the endpoint level *in vivo* data and POD level *in vivo* data following the methods outlined in Fig 1, step 7–8. Following those methods for the example results in Fig 3, in the forward dosimetry comparison a win would be counted for the PBTK result while, in a separate set of counts for reverse dosimetry, a win would be counted for AC_50_. Fig 3 included values for 1 of the 10 sets of random results, wherein the TK were randomized. In the following results, comparisons were made for each separate set of random results, so that a total of 10 sets of comparisons were made. Medians and standard deviations were then determined from these counts.

**Fig 6:**
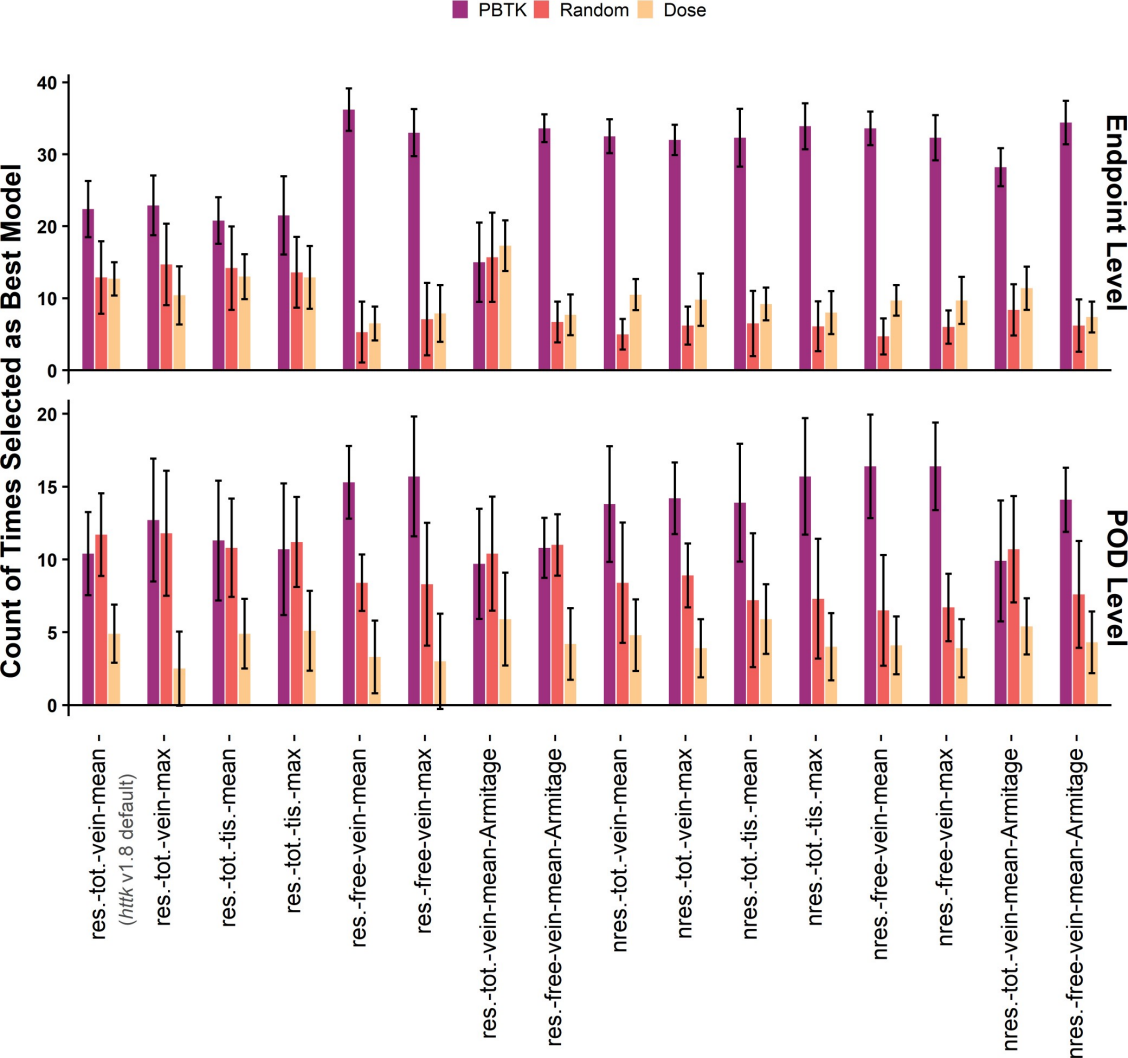
Allocated counts from the forward dosimetry method. Counts compare *in vitro* AC_50_ with predicted *in vivo* concentration for the endpoint level analysis (top row) and POD level analysis (bottom row) as a function of the assumptions used in application of the PBTK model. Counts are from assay-effect pairs with at least 20 unique chemicals and are median values from the 10 sets of comparisons. The error bars are plus or minus two standard deviations from the 10 comparisons. Labels on the x-axis indicate assumption set: for clearance (res.–restrictive, nres.–nonrestrictive), *in vivo* concentration selection (tot.–total, free, vein, tis.–tissue, mean, max), and use of the Armitage disposition model to estimate the free concentration *in vitro*.

**Fig 7:**
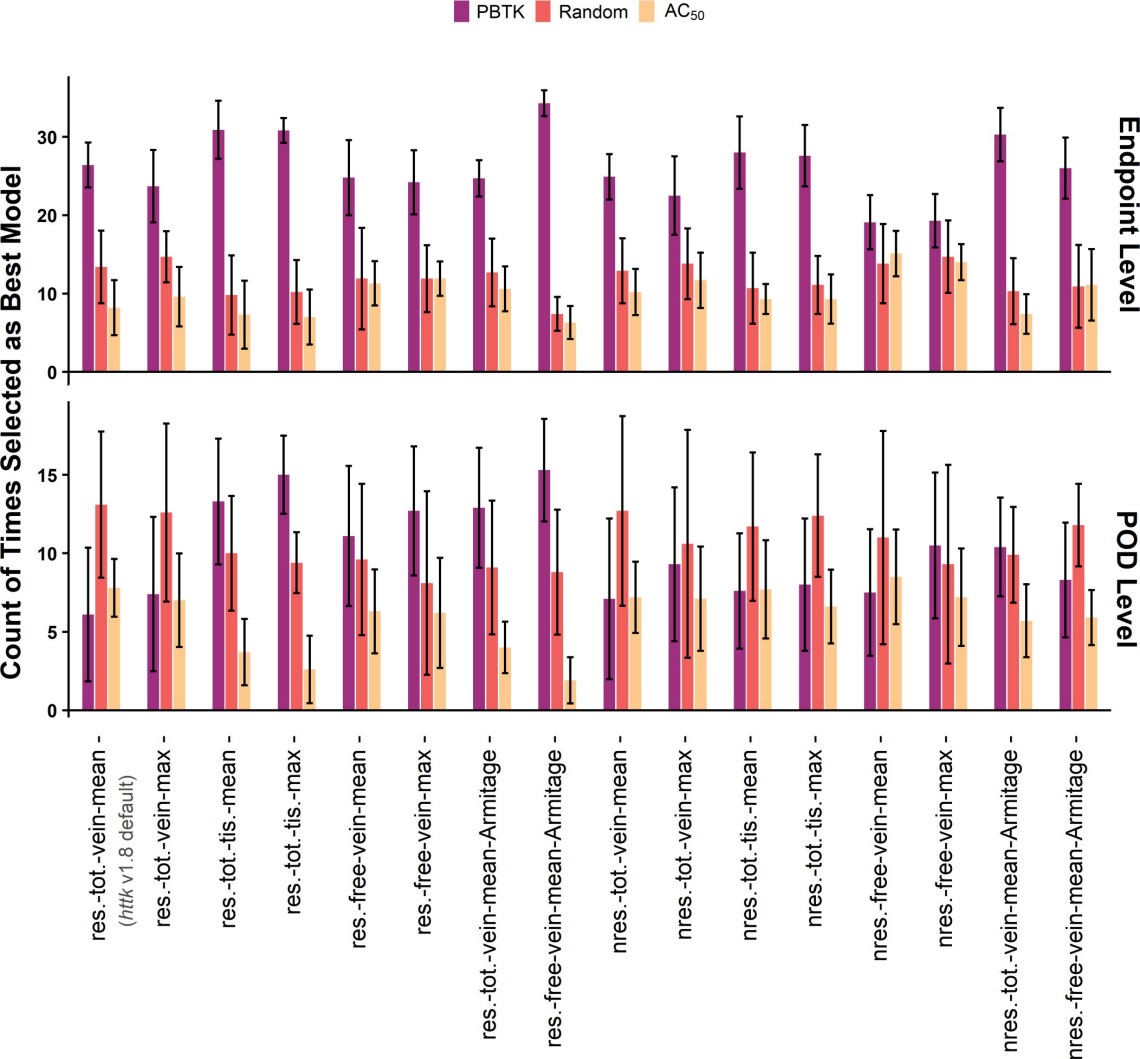
Allocated counts from the reverse dosimetry method. Counts compare *in vivo* dose with predicted AED from *in vitro* toxicity assay results for the endpoint level analysis (top row) and POD level analysis (bottom row) as a function of the assumptions used in application of the PBTK model. Counts are from assay-effect pairs with at least 20 unique chemicals and are median values from the 10 sets of comparisons. The error bars are plus or minus two standard deviations from the 10 comparisons. Labels on the x-axis indicate assumption set: for clearance (res.–restrictive, nres.–nonrestrictive), *in vivo* concentration selection (tot.–total, free, vein, tis.–tissue, mean, max), and use of the Armitage disposition model to estimate the free concentration *in vitro*.

The results of applying the analyses across all combinations of assumptions and concentration selection are shown in Figs 6 and 7 for comparisons based on at least 20 different chemicals. Figs 6 and 7 represent the results of more than 15,600 comparisons across assumptions, *in vivo* endpoints (for the endpoint level analysis), and *in vitro* component endpoints. The assumptions evaluated include clearance (restrictive and nonrestrictive), internal concentration selection (mean or max; total venous plasma, free venous plasma, or tissue), and estimation of the free concentration in the *in vitro* bioactivity assay using the model by Armitage et al. [51]. Results for comparisons based on at least 5 different chemicals are available in S1 Fig and S2 Fig. In the endpoint level analysis, study types were evaluated separately and then the counts were compiled in Figs 6 and 7. A breakdown of the results for the endpoint level analysis by study type is available in S3 Fig, S4 Fig, S5 Fig, and S6 Fig, and these results exhibit similar trends. Across all comparisons, minimal differences were observed between selection of the mean or maximum *in vivo* concentration. As such, the results for use of the Armitage *in vitro* disposition model are only shown in combination with the mean venous plasma concentration.

For the forward dosimetry comparison (Fig 6), the endpoint level analysis suggests that in general, using the PBTK model for IVIVE better clarifies the association between *in vitro* bioactivity and *in vivo* toxicity data than either the random result and untransformed dose. Improvements in performance appear greater when nonrestrictive clearance is assumed, or when the free plasma concentration is used with restrictive clearance. Utilizing the Armitage model to estimate the free *in vitro* concentration performs well when combined with the selection of the *in vivo* mean free venous plasma concentration (res.-free-vein-mean-Armitage). Similar trends are observed in the POD level analysis, although the differences between counts are smaller. For the reverse dosimetry comparisons shown in Fig 7, the trends in the endpoint level analysis differ from those observed for forward dosimetry. The counts for PBTK with restrictive clearance are slightly higher relative to those with nonrestrictive clearance, while the highest counts are achieved when comparisons are made using the *in vivo* mean free venous plasma concentration in combination with the *in vitro* free concentration predicted by the Armitage model (res.-free-vein-mean-Armitage). For the reverse dosimetry POD level analysis, the random result produced higher counts for certain assumptions, but these exhibit large deviations.

In general, the PBTK results performed better for the endpoint level analyses than the POD level analyses. For the endpoint level analysis comparisons versus assay results were made for specific *in vivo* endpoints and study types, whereas for the POD level analysis doses were taken as the minimum across study types and pathology for a given chemical. It would be expected that trends may be stronger across a specific endpoint as opposed to comparisons across different pathologies and different study types, as in the POD level analyses. Separately from the determination of ORMSE, variances for log_10_ transformed AC_50_ as well as variances for log_10_ transformed dose were determined for each comparison made in the two separate analyses for an arbitrary assumption set. Table 1 shows the medians of these variances determined for all comparisons of *in vitro* assay endpoint and *in vivo* endpoint (endpoint level analysis) and comparisons of *in vitro* assay endpoints to POD data (POD level analysis). While the variance in AC_50_ was similar in either analysis, the variance in dose was larger for the POD level.

**Table 1:** Median of variances for log_10_ transforms of AC_50_ and dose by analysis level.

	AAC_50_	DDose
Endpoint Level	00.21	00.46
POD Level	00.17	00.77

**Fig 8:**
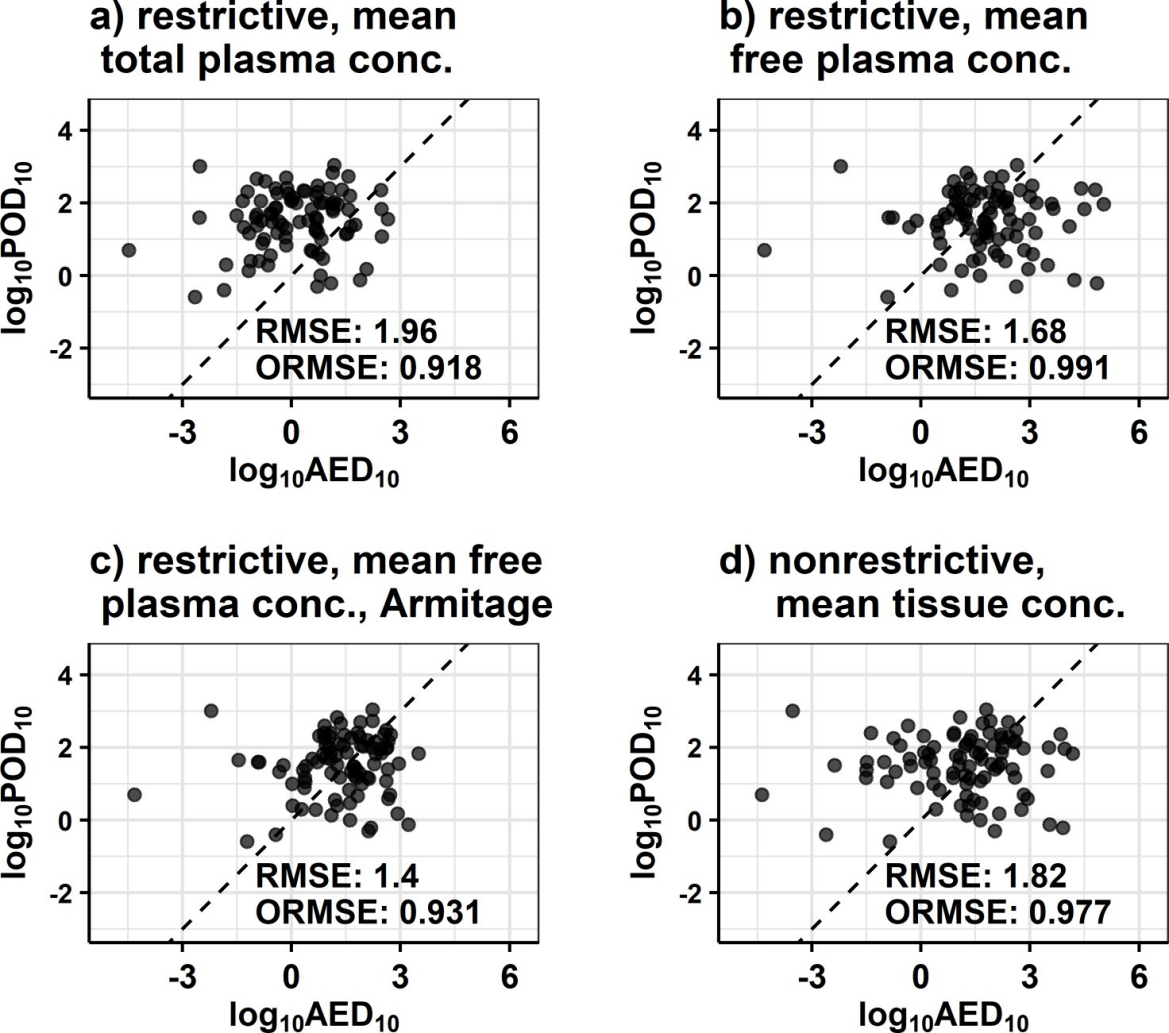
Plots of the log_10_ transforms of the 10^th^ percentile dose from POD level *in vivo* data vs the 10^th^ percentile AED using the PBTK model, determined with a time scale of the corresponding lowest dose. Each point corresponds to a particular chemical. Results are for the assumption set of total mean concentration, and are otherwise indicated by the panel labels: a) restrictive clearance with *in vivo* mean total venous plasma concentration, b) restrictive clearance with *in vivo* mean free venous plasma concentration, c) restrictive clearance with *in vivo* mean free venous plasma and free concentration *in vitro* predicted by the Armitage model, d) nonrestrictive clearance with mean tissue concentration. The dashed lines are y = x lines. Corresponding RMSE and ORMSE (the latter defined for the standardized variables) are also reported.

PBTK performs better than the randomized result and the untransformed result in general for the endpoint level analysis. However, given the limitations of the available chemical-specific data, an optimal set of assumptions for IVIVE among those evaluated in Figs 6 and 7 cannot be clearly identified. The assumptions that appear to perform best include restrictive clearance with free mean or free max venous plasma concentration, nonrestrictive clearance with total mean or max tissue concentration, and restrictive free mean venous plasma concentration *in vivo* combined with estimation of the free concentration *in vitro* by the Armitage model. Prior work [27] has been based on only one set (*httk* v1.8) of these assumptions (restrictive clearance; total steady-state concentration which is analogous to the total mean concentration used here) which has been shown to be slightly inferior to other assumptions for forward-dosimetry in Fig 6 but more equivocal for reverse-dosimetry (Fig 7) and remains a plausible option. Using those set of conditions, Wetmore, *et al*. demonstrated the potential to use TK for IVIVE to compare lowest *in vivo* doses with the lowest AED estimated from *in vitro* bioactivity data [27]. Both *httk* v1.8 [23] and Wetmore et al. [20] include a correction by Kilford et al. [37] for the fraction of chemical bound in the *in vitro* clearance assays [55]. Analogous comparisons produced in our current work are shown in Fig 8 for four of the assumption sets. The log_10_ transforms of the lower 10^th^ percentile POD (POD_10_) per chemical vs the lower 10^th^ percentile AED (AED_10_) predicted from the *in vitro* toxicity assay are plotted with assumptions as indicated in the titles of the sub-plots. Corresponding RMSE of the log_10_ transformed variables and ORMSE of the standardized log_10_ transformed variables are reported. Additional figures for the other assumption sets and mean concentration are available in S7 Fig. In this exercise, it would be desirable for dose and AED to be highly associated and for AED to be less than the corresponding dose, the argument being that the *in vitro* assays should be more sensitive to the initial perturbation that may cause an adverse effect. Using the *in vivo* mean free venous plasma concentration with the free *in vitro* concentration from the Armitage model (Fig 8D) has a significantly lower RMSE. While this might be preferable in terms of predicting the mean, the restrictive clearance results using mean total plasma concentration (8a) are more conservative (i.e., they rarely underestimate the POD_10_). Additionally, in this exercise Fig 8A has a slightly lower ORMSE, indicating a potentially stronger association despite a greater RMSE. Lower RMSE alone suggests improved accuracy of the mean, but in this case does not correspond with improvements in association.

**Fig 9:**
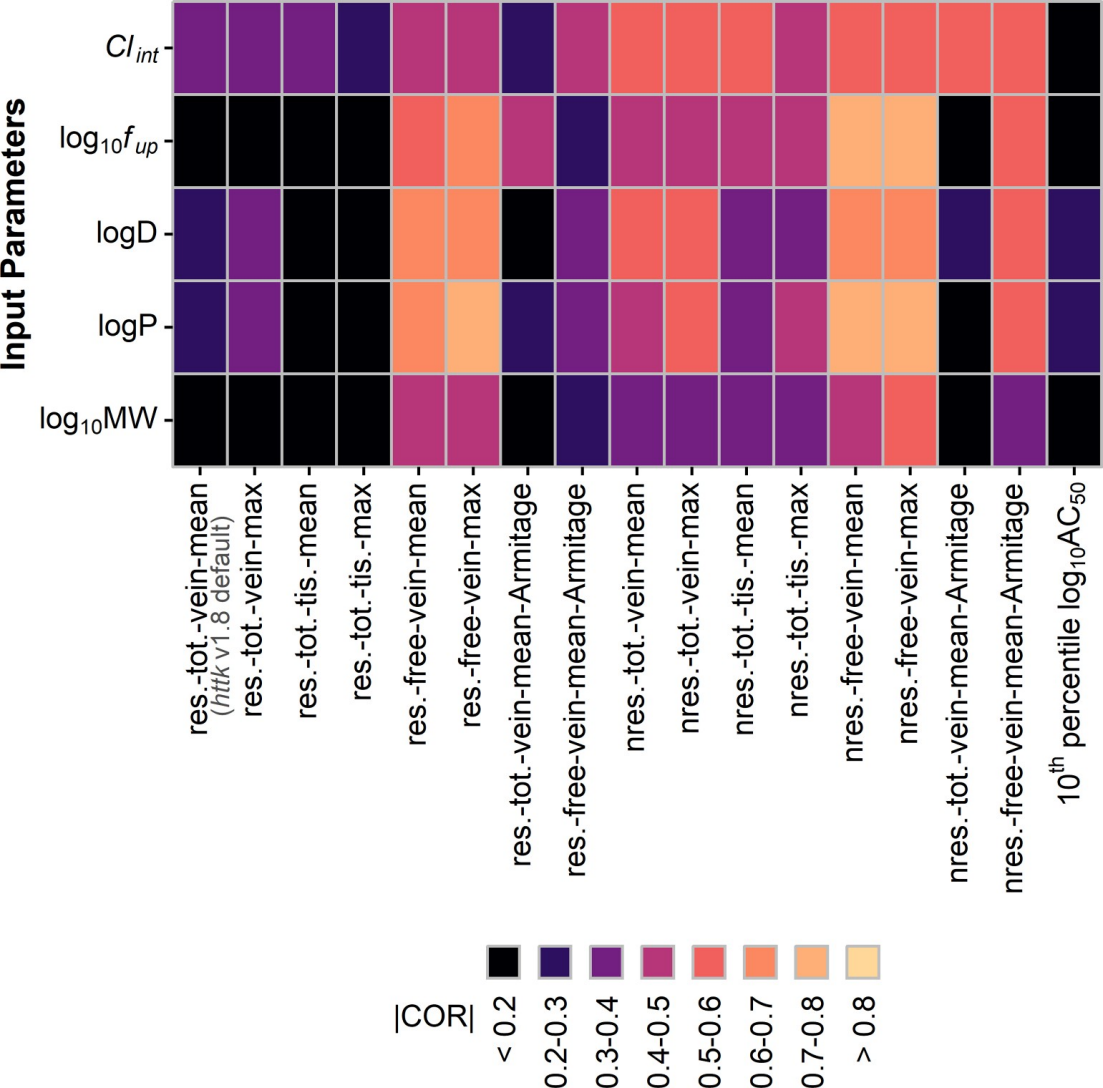
Heatmap of correlation between residuals and input parameters. Residuals are from comparisons of log_10_ POD_10_ and log_10_ AED_10_ for each assumption set. The absolute value of the Pearson’s correlation coefficient (|COR|) was determined between the residuals and each input parameter. Labels on the x-axis indicate assumption set: for clearance (res.–restrictive, nres.–nonrestrictive), concentration selection (tot.–total, free, vein, tis.–tissue, mean, max), use of the Armitage disposition model to estimate the free concentration *in vitro*, and direct comparison to 10^th^ percentile AC_50_ values.

Lastly, it is useful to understand if model performance depends on the type of chemical. As a simple evaluation, we can examine the association between physical chemical properties and residual error. Importantly, this also serves as an evaluation of model bias. The models in this work are based on input parameters including *f*_*up*_, *Cl*_*int*_, the octanol-water partition coefficient (logP), the dissociation constant (logD), and molecular weight (MW). As a final test, we evaluate the association between these parameters and the residuals from the comparison of the log10 transforms of POD_10_ vs AED_10_ for each model. For this test, we use the absolute value of the Pearson’s correlation coefficent, |COR|, separately determined between the residuals and each parameter. Results are represented by the heatmap shown in Fig 9, where brighter colors correspond to higher values for |COR|. Under ideal circumstances, the model should accurately account for the relationship between the chemical, the parameter, and the outcome (AED_10_) such that |COR| should be low. However, Fig 9 shows that |COR| is high for certain combinations of model and input parameter. Comparisons relative to the 10^th^ percentile log_10_ AC_50_ values are included for reference. For assumptions that performed poorly in previous tests, we generally see little correlation with the different input parameters. This is true for the base set of assumptions of restrictive clearance with mean total venous plasma concentration. However, the residuals for those models that performed well appear to exhibit some dependence on the input parameters. For the mean or max free *in vivo* plasma concentration with restrictive clearance, the residuals appear to be highly correlated with *f*_*up*_, logP, and logD. This suggests that the model using these assumptions may not fully account for the differences in binding between the *in vitro* and *in vivo* systems. However, using the Armitage model to estimate the free concentration *in vitro* has lower correlation between the residuals and the parameters of *f*_*up*_, logP, and logD. Using nonrestrictive clearance exhibits higher values for |COR|, particularly for *Cl*_*int*_, across the different combinations of assumptions. This holds for nonrestrictive clearance with mean or max tissue concentration.

## Discussion

Understanding our ability to use *in vitro* and *in silico* methods to quantitatively predict known doses exhibiting pathological effects *in vivo* is a prerequisite to estimating toxic doses for chemicals without *in vivo* toxicological data. In this work, the application of a PBTK model to clarify the association between *in vitro* bioactivity and *in vivo* toxicity data [11, 13, 27] was evaluated across a broad range of chemicals, *in vitro* assays, *in vivo* endpoints, and modeling assumptions. Evaluations were carried out for two analysis levels: 1) each specific *in vivo* endpoint was compared with each *in vitro* high-throughput screening assay component endpoint and 2) POD determined across pathologies and study types were compared with each *in vitro* high-throughput screening assay component endpoint. For both analysis levels (endpoint level and POD level, respectively), results were compared for forward dosimetry and reverse dosimetry. In the endpoint level analysis, both the forward and reverse dosimetry results demonstrated an improved performance when using the PBTK model. This strongly suggests that applying toxicokinetic models elucidates the association between *in vitro* bioactivity and *in vivo* toxicity data, particularly when study type and specific effect are considered.

The analyses presented here were designed to clarify the association between *in vitro* bioactivity and *in vivo* toxicity data. Results are not meant to suggest prediction of a particular *in vivo* endpoint by a specific *in vitro* assay component endpoint. Rather, improved performance in general indicates that incorporating TK would be beneficial when extrapolating quantitative results from models that rely on an ensemble of assay results, such as the estrogen receptor pathway model of Judson et al. that used 18 different ToxCast assays [8]. Furthermore, *in vitro* bioactivity and *in vivo* toxicity data in this work were restricted to positive only results. While TK may suggest that an AED associates with an *in vivo* dose, TK will not convert a negative assay hit to a positive one or vice-versa. Although there was a general observation of improved performance for the PBTK model in the endpoint level analysis, there was dissimilarity in the trends observed with respect to the different assumption sets. There were also differences in the results between the endpoint level and POD level analyses. The ambiguity of the trends with respect to association (i.e., Figs 6 and 7) when examining various assumptions and dose metrics suggested the potential of a range of possible results in different applications of TK. In some cases, untransformed *in vivo* dose was associated with *in vitro* AC_50_ (nonzero counts for dose and AC_50_ in Figs 6 and 7). This may be a reflection of the fact that doses are often selected to reflect the amount of chemical that can be tolerated by the test animal [56]. Additionally, the relatively higher counts for random in the POD level analysis may be due to the selection of minimum AED. We did observe, however, that using TK yielded a stronger association more often than it did not. Finally, subsequent analysis comparing residuals with input parameters suggested that several of the model assumptions had certain pronounced biases. Collectively, utilizing restrictive clearance with the mean free venous plasma concentration and estimation of free concentration *in vitro* using the Armitage model appeared to perform slightly better in terms of 1) the ability to clarify the association between *in vitro* bioactivity and *in vivo* data (Figs 6 and 7), 2) accuracy (i.e., RMSE) of AED_10_ extrapolation relative to POD_10_ (Fig 8), and 3) relatively lower dependence on input parameters (Fig 9).

### The metrics evaluated

The ORMSE of the standardized log_10_ transforms was selected as the statistical parameter for the basis of the comparisons made in this work to 1) enable comparison between forward and reverse dosimetry, 2) allow comparison across data with different units, and 3) elucidate the association between the *in vitro* bioactivity and *in vivo* toxicity data. As in the example regression in Fig 3, the ORMSE may be sensitive to outliers. However, as we carried out the analysis across a range of assay endpoints, *in vivo* effects, and chemicals, we expect this effect was moderated, as evidenced by the general observation of improved performance with the PBTK result for the endpoint level analysis. Furthermore, we also used ten sets of randomized results as additional references for comparison of the performance of the application of the PBTK model. If the performance across all *in vitro* and *in vivo* comparisons were due to small differences in ORMSE, then the randomized result would perform similarly to the PBTK result. However, this was not observed to be the case for the endpoint level analysis. Standard parameters such as R^2^ and RMSE are y-oriented and would be less comparable between forward and reverse dosimetry. Evaluating the total sum of squares of the residuals would be dimensional and would not necessarily be relatable to the strength of an association. Another alternative to using RMSE would be to represent the uncertainty in the measured *in vitro* bioactivity and *in vivo* toxicity data via bootstrapping or another methodology, but this would require an *a priori* understanding of the uncertainty at different levels of the data.

As the example regression in Fig 3 showed, the comparison of AC_50_ and C_PBTK_ in the forward dosimetry result is separate from the comparison of *in vivo* dose and AED_*PBTK*_ in the reverse dosimetry result. From Eq 1, we see that the values of C_PBTK_ and AED_PBTK_ are inversely related, that C_PBTK_ is a function of the dose and the study length (i.e. dosing regimen), and that AED_PBTK_ is a function of AC_50_ and the corresponding study length of interest. The residuals of the log_10_ transforms are therefore equal and opposite (Eqs 7 and 8).

$$\log_{10}\;AC_{50,j}-\log_{10}\;C_{PBTK,j}=residual_{j}$$ (7)

$$\log_{10}\;dose_{j}-\log_{10}\;AED_{PBTK,j}=-residual_{j}$$ (8)

As such, the total sum of squares would be equivalent for both comparisons. However, any statistic that accounts for the distribution of the variables (e.g., the variance, ORMSE of standardized variables) will likely differ. In this work, we are more concerned with the latter as our focus is on association. While related, *C*_*PBTK*_ and AED_*PBTK*_ will produce separate sets of results in their comparisons, unless the residuals between variables approach zero. Importantly, the basic relationship between dose and concentration suggests the need to compare results for both forward and reverse dosimetry when evaluating any model applied for quantitative IVIVE.

### The influence of the evaluated assumptions

**Fig 10:**
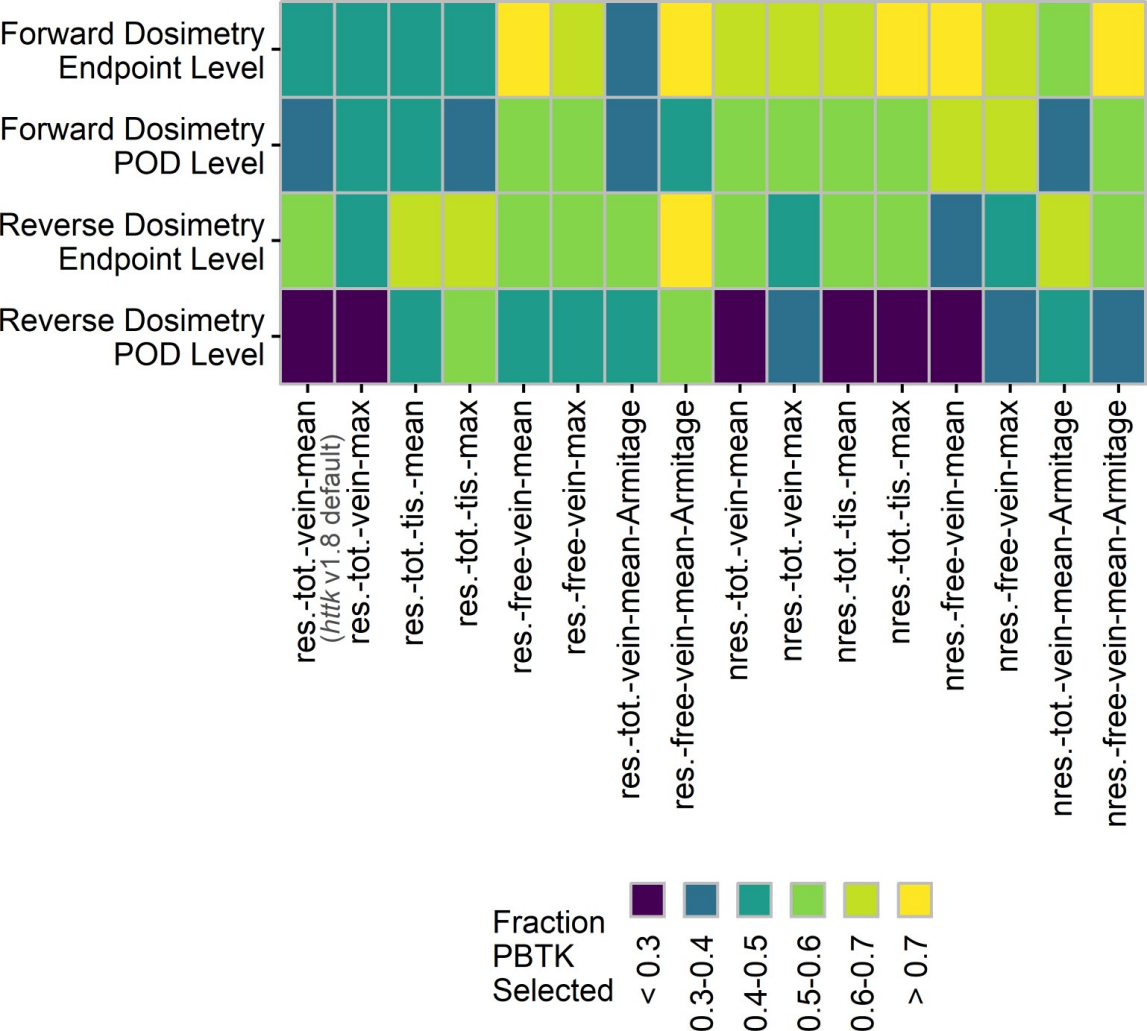
Heatmap summarizing results previously shown in Figs 6 and 7. The fraction of counts for which the PBTK model was selected is indicated by color, with brighter shades indicating a higher fraction of selection. Labels on the x-axis indicate assumption set: for clearance (res.–restrictive, nres.–nonrestrictive), concentration selection (tot.–total, free, vein, tis.–tissue, mean, max), and use of the Armitage disposition model to estimate the free concentration *in vitro*.

Although this work demonstrates the potential of TK to elucidate the association between *in vitro* bioactivity and *in vivo* toxicity data, it was unclear which specific set of assumptions in the application of TK for IVIVE yielded an optimal result in Figs 6 and 7. For ease of reference, the results in Figs 6 and 7 are summarized in Fig 10 by the fraction that the PBTK result was selected over the random result and untransformed values (i.e., ratio of counts for PBTK in Figs 6 and 7 to the corresponding total count per dosimetry, analysis level, and assumption set). Of the assumptions evaluated, selection of either the mean or maximum *in vivo* concentration seemed to have little influence on performance. While there were clear differences between the results for the other layers of the assumptions, trends in the forward dosimetry results were not always corroborated by the reverse dosimetry results. Those sets of assumptions that consistently performed well include restrictive clearance with free venous plasma concentration, restrictive clearance with free venous plasma *in vivo* concentration combined with free *in vitro* concentration predicted by the Armitage model, and nonrestrictive clearance with tissue concentration.

Free concentration *in vivo* is generally assumed to be the relevant concentration for effecting a biological response; however, this should be specific to the local active site and as such may be intracellular and subject to active transport or other processes [57]. In this work, we have only evaluated the free concentration in venous plasma, in part due to the perfusion limited assumption of our model. Furthermore, we have only evaluated the free (aqueous phase) *in vitro* concentration as many of the evaluated assays are not cell-based. Even for comparison with a given assay endpoint, the relative prevalence of various types of binding sites *in vivo* and *in vitro* may differ and result in differences in the fraction of free chemical. Despite these qualifications, using the free *in vivo* concentration with restrictive clearance and the nominal *in vitro* concentration performed reasonably well, likely because many of the *in vitro* assays evaluated are not cell-based and have limited material available for binding. A more accurate result, particularly for cell-based assays, may be expected if the *in vitro* concentration were corrected for nonspecific binding by using an *in vitro* disposition model. For restrictive clearance, this expectation was corroborated by our results which demonstrated improved performance when using free *in vivo* concentration in combination with the Armitage model to estimate free *in vitro* concentration [51]. Furthermore, the analysis of residuals (Fig 9) showed that using the free *in vivo* concentration alone exhibited bias in the results with respect to binding related parameters of *f*_*up*_, logP, and logD. However, this bias appeared to be reduced when using free *in vivo* concentration and free *in vitro* concentration.

This differs from the conclusions proposed in the work by Casey et al. [58], which evaluated the use of TK to extrapolate the ER pathway model [8] and compared results relative to OECD guideline uterotrophic *in vivo* data. Based on direct comparison of log_10_ RMSE between models, the authors concluded that using either a steady state free plasma concentration (*in vivo*) or the *in vitro* intracellular concentration predicted by the Armitage model provided a more accurate result for this specific pathway. However, lower RMSE indicates improved accuracy of the mean, but does not necessarily imply a stronger association or that the model is more informative. Our results instead suggest the possibility that using the mean free plasma concentration with restrictive clearance is one of several options exhibiting mild improvements in association over other model assumptions/selections when applied across a broad range of *in vitro* and *in vivo* data. Furthermore, we observed additional improvement when using free *in vivo* concentration in combination with free *in vitro* concentration, which might be considered a theoretically more justifiable comparison.

Nonrestrictive clearance with tissue concentration also appeared to perform well in terms of clarifying the association between *in vitro* bioactivity and *in vivo* toxicity data (Fig 10). However, high values for |COR| were observed for several input parameters (Fig 9), particularly with respect to *Cl*_*int*_. This trend was observed for other combinations of assumptions with nonrestrictive clearance. As such, the results call into question the suitability of using nonrestrictive clearance as a broad assumption. It should be noted that restrictive and nonrestrictive clearance are the two limits of an equilibrium assumption made to simplify dynamic binding, mass transport, and reaction processes that occur during hepatic clearance. Further understanding of the dependence of the rates of those processes on the chemical may provide more representative results.

### Implications for future work

The results of this work demonstrate that TK improves our ability to elucidate the association between *in vitro* bioactivity and *in vivo* toxicity data when evaluations are made per specific *in vivo* endpoints and study types. Therefore, *in vitro* bioactivity results should be translated using TK for IVIVE when developing machine learning and other statistical models for predicting *in vivo* toxicity. This potential suggests that improvements to the understanding of TK for environmental chemicals would be beneficial to enabling new approach methods. In this work, the assumptions were evaluated based on application across all the data; there was no selection made based on the particular assay or *in vivo* effect. In general, it appeared that using restrictive clearance with free *in vivo* venous plasma concentration and free *in vitro* concentration performed well. Although this was somewhat less conservative than the base set of assumptions (restrictive clearance with mean total plasma concentration, Fig 8A), if uncertainty were extrapolated it may be reasonable to select a lower bound (e.g., the 5^th^ percentile) as a more conservative estimate of POD. Additionally, for certain *in vitro* assays or *in vivo* endpoints, some assumptions may work better than others, particularly with respect to concentration selection and use of an *in vitro* disposition model. Variation of those assumptions based on relevancy to the assay in question may be reasonable. Similarly, it may be possible to vary certain assumptions based on chemical. This may particularly be applicable to nonrestrictive and restrictive clearance, which should vary by chemical.

We have shown that PBTK clarifies the association between *in vitro* screening assays and *in vivo* toxicity. Using restrictive clearance with free *in vivo* venous plasma concentration and free *in vitro* concentration appeared to perform as well as or better than other model assumptions. While in that case the residual error was somewhat less dependent on the input parameters, there remained some bias–this suggests the potential for model improvement. Based on the performance afforded by PBTK, other methods to further improve the accuracy and understanding of TK should be pursued [59], particularly with respect to chemical-specific TK parameters. For example, this work assumed 100% fraction absorbed in the gut and no gut metabolism–a better understanding of oral bioavailability would be beneficial. Differences were observed in the performance of restrictive and nonrestrictive clearance, although they were not consistent between forward and reverse dosimetry. Restrictive and nonrestrictive clearance are two limits of a potential range of off-binding rates of an absorbed chemical from protein. Where available, *in vitro* methods could be used to measure these and other toxicokinetic parameters. Further evaluation of both forward and reverse dosimetry is important to fully understand the relationship between *in vitro* bioactivity and *in vivo* toxicity data.

This work evaluated applying toxicokinetics for rat to predict doses for which *in vivo* effects may be observed in rat. The PBTK model used here can be scaled to human by using human physiological parameters and human specific *in vitro* or *in silico* determined toxicokinetic parameters (i.e., *Cl*_*int*_ and *f*_*up*_). Human specific data have already been collected for hundreds of chemicals [12, 19]. Thus, the IVIVE principles evaluated here may be applied to humans. Ultimately, AED for humans can then be compared with predicted rates of exposure to provide an estimate of chemical risk [2, 5, 17–21].

## Supporting information

S1 Fig Allocated counts from forward dosimetry for comparisons with at least 5 chemicals. Counts compare *in vitro* AC_50_ with predicted *in vivo* concentration for the endpoint level analysis (top row) and POD level analysis (bottom row) as a function of the assumptions used in application of the PBTK model. Counts are from *in vivo-in vitro* pairs with at least 5 unique chemicals and are median values from the 10 sets of comparisons. The error bars are plus or minus two standard deviations from the 10 comparisons. Labels on the x-axis indicate assumption set: for clearance (res.–restrictive, nres.–nonrestrictive), concentration selection (tot.–total, free, vein, tis.–tissue, mean, max), and use of the Armitage disposition model to estimate the free concentration *in vitro*.
(TIFF)

S2 Fig Allocated counts from reverse dosimetry for comparisons with at least 5 chemicals. Counts compare *in vivo* dose with predicted AED from *in vitro* toxicity assay results for the endpoint level analysis (top row) and POD level analysis (bottom row) as a function of the assumptions used in application of the PBTK model. Counts are from *in vivo-in vitro* pairs with at least 5 unique chemicals and are median values from the 10 sets of comparisons. The error bars are plus or minus two standard deviations from the 10 comparisons. Labels on the x-axis indicate assumption set: for clearance (res.–restrictive, nres.–nonrestrictive), concentration selection (tot.–total, free, vein, tis.–tissue, mean, max), and use of the Armitage disposition model to estimate the free concentration *in vitro*.
(TIFF)

S3 Fig Allocated counts from forward dosimetry for comparisons with at least 5 chemicals by study type. Counts compare *in vitro* AC_50_ with predicted *in vivo* concentration for the endpoint level analysis (top row) and POD level analysis (bottom row) as a function of the assumptions used in application of the PBTK model. Counts are from *in vivo-in vitro* pairs with at least 5 unique chemicals and are median values from the 10 sets of comparisons. The error bars are plus or minus two standard deviations from the 10 comparisons. Labels on the x-axis indicate assumption set: for clearance (res.–restrictive, nres.–nonrestrictive), concentration selection (tot.–total, free, vein, tis.–tissue, mean, max), and use of the Armitage disposition model to estimate the free concentration *in vitro*. Results are separated by study type: chronic (CHR), subchronic (SUB), and developmental (DEV).
(TIFF)

S4 Fig Allocated counts from reverse dosimetry for comparisons with at least 5 chemicals by study type. Counts compare *in vivo* dose with predicted AED from *in vitro* toxicity assay results for the endpoint level analysis (top row) and POD level analysis (bottom row) as a function of the assumptions used in application of the PBTK model. Counts are from *in vivo-in vitro* pairs with at least 5 unique chemicals and are median values from the 10 sets of comparisons. The error bars are plus or minus two standard deviations from the 10 comparisons. Labels on the x-axis indicate assumption set: for clearance (res.–restrictive, nres.–nonrestrictive), concentration selection (tot.–total, free, vein, tis.–tissue, mean, max), and use of the Armitage disposition model to estimate the free concentration *in vitro*. Results are separated by study type: chronic (CHR), subchronic (SUB), and developmental (DEV).
(TIFF)

S5 Fig Allocated counts from forward dosimetry for comparisons with at least 20 chemicals by study type. Counts compare *in vitro* AC_50_ with predicted *in vivo* concentration for the endpoint level analysis (top row) and POD level analysis (bottom row) as a function of the assumptions used in application of the PBTK model. Counts are from *in vivo-in vitro* pairs with at least 20 unique chemicals and are median values from the 10 sets of comparisons. The error bars are plus or minus two standard deviations from the 10 comparisons. Labels on the x-axis indicate assumption set: for clearance (res.–restrictive, nres.–nonrestrictive), concentration selection (tot.–total, free, vein, tis.–tissue, mean, max), and use of the Armitage disposition model to estimate the free concentration *in vitro*. Results are separated by study type: chronic (CHR), subchronic (SUB), and developmental (DEV).
(TIFF)

S6 Fig Allocated counts from reverse dosimetry for comparisons with at least 20 chemicals by study type. Counts compare *in vivo* dose with predicted AED from *in vitro* toxicity assay results for the endpoint level analysis (top row) and POD level analysis (bottom row) as a function of the assumptions used in application of the PBTK model. Counts are from *in vivo-in vitro* pairs with at least 20 unique chemicals and are median values from the 10 sets of comparisons. The error bars are plus or minus two standard deviations from the 10 comparisons. Labels on the x-axis indicate assumption set: for clearance (res.–restrictive, nres.–nonrestrictive), concentration selection (tot.–total, free, vein, tis.–tissue, mean, max), and use of the Armitage disposition model to estimate the free concentration *in vitro*. Results are separated by study type: chronic (CHR), subchronic (SUB), and developmental (DEV).
(TIFF)

S7 Fig Plots of the log10 transforms of the 10^th^ percentile dose from POD level *in vivo* data vs the 10^th^ percentile AED using the PBTK model, determined with a time scale of the corresponding lowest dose. Each point corresponds to a particular chemical. Results are for the assumption set of total mean concentration, and are otherwise indicated by the panel labels: a) restrictive clearance with *in vivo* mean free total venous plasma concentration, b) restrictive clearance with *in vivo* mean venous plasma concentration and free concentration *in vitro* predicted by the Armitage model, c) nonrestrictive clearance with *in vivo* mean total plasma concentration, d) nonrestrictive clearance with *in vivo* mean free venous plasma concentration, e) nonrestrictive clearance with *in vivo* mean total venous plasma concentration and free concentration *in vitro* predicted by the Armitage model, f) nonrestrictive clearance with *in vivo* mean free venous plasma concentration and free concentration *in vitro* predicted by the Armitage model. The dashed lines are y = x lines. Corresponding RMSE and ORMSE (the latter defined for the standardized variables) are also reported.
(TIFF)

S1 File Analysis scripts and input data. (ZIP)

S2 File List of abbreviations. (DOCX)

S3 File List of assumptions evaluated. (DOCX)

S1 Table Measured *Cl_int_* and *f_up_* for rat. (CSV)

S2 Table GC-MS methods. (XLSX)

S3 Table LC-MS methods. (XLSX)

S4 Table Endpoint level *in vivo* toxicity data for rat. (CSV)

S5 Table POD level *in vivo* toxicity data for rat. (CSV)

S6 Table *In vivo* endpoints evaluated in this work. (CSV)
