# Supplementary figures and images for: Using the concordance of in vitro and in vivo data to evaluate extrapolation assumptions
Source: PLoS One. 2019 May 28;14(5):e0217564. doi: 10.1371/journal.pone.0217564 (PMC6538186; doi:10.1371/journal.pone.0217564)

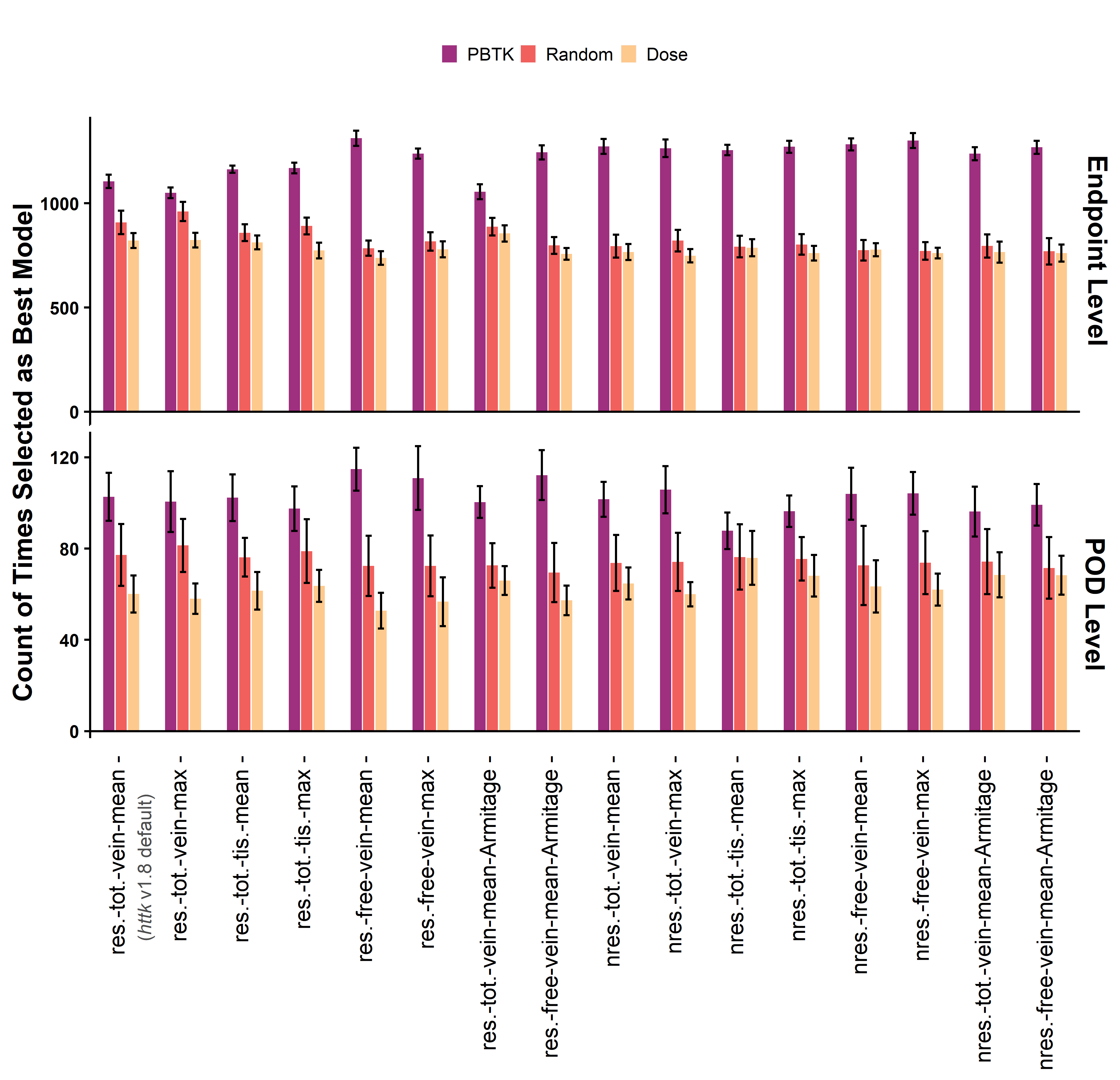

Supplement: S1 Fig — Counts compare in vitro AC50 with predicted in vivo concentration for the endpoint level analysis (top row) and POD level analysis (bottom row) as a function of the assumptions used in application of the PBTK model. Counts are from in vivo-in vitro pairs with at least 5 unique chemicals and are median values from the 10 sets of comparisons. The error bars are plus or minus two standard deviations from the 10 comparisons. Labels on the x-axis indicate assumption set: for clearance (res.–restrictive, nres.–nonrestrictive), concentration selection (tot.–total, free, vein, tis.–tissue, mean, max), and use of the Armitage disposition model to estimate the free concentration in vitro. (TIFF) [file pone.0217564.s001.tiff]

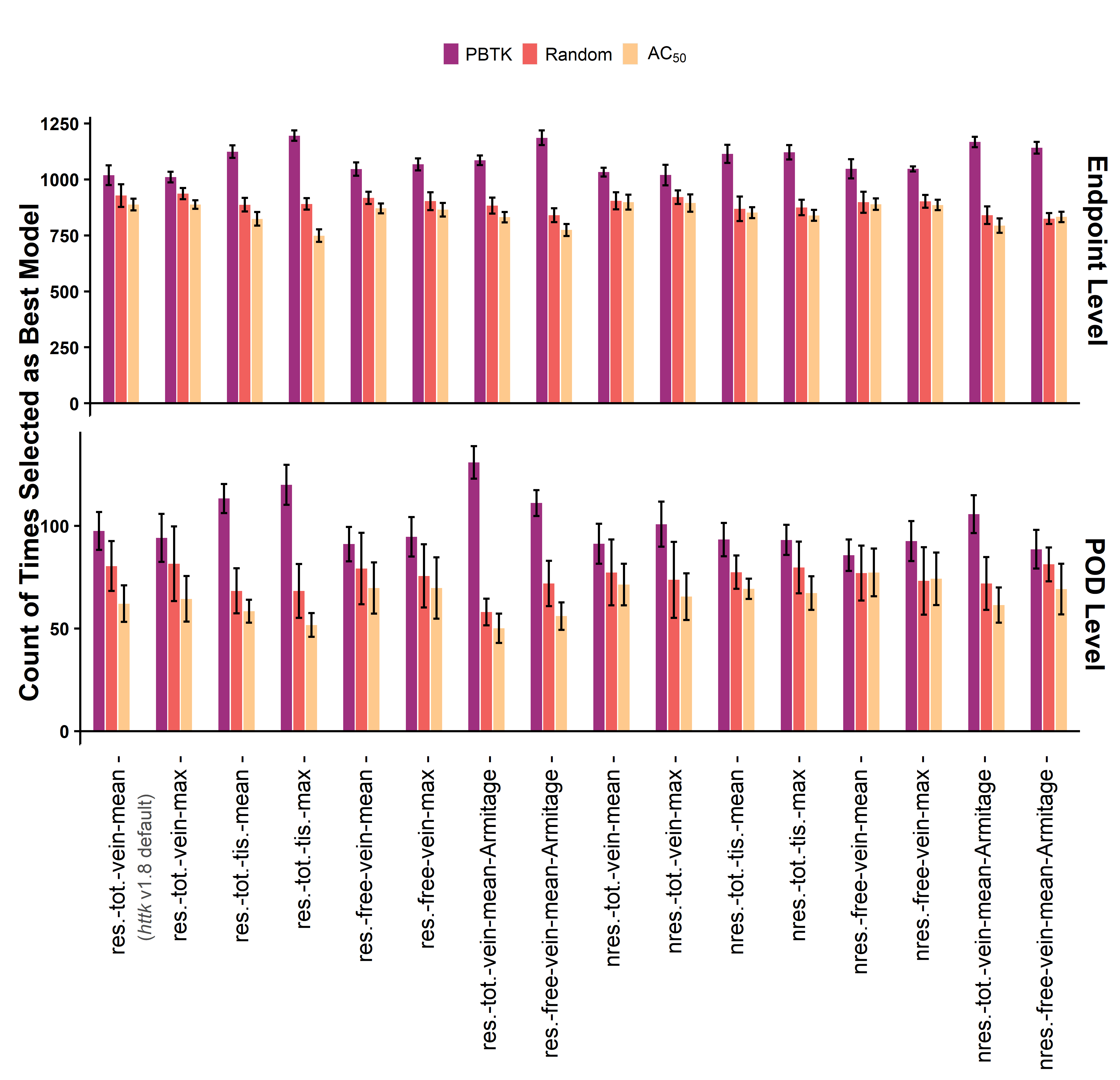

Supplement: S2 Fig — Counts compare in vivo dose with predicted AED from in vitro toxicity assay results for the endpoint level analysis (top row) and POD level analysis (bottom row) as a function of the assumptions used in application of the PBTK model. Counts are from in vivo-in vitro pairs with at least 5 unique chemicals and are median values from the 10 sets of comparisons. The error bars are plus or minus two standard deviations from the 10 comparisons. Labels on the x-axis indicate assumption set: for clearance (res.–restrictive, nres.–nonrestrictive), concentration selection (tot.–total, free, vein, tis.–tissue, mean, max), and use of the Armitage disposition model to estimate the free concentration in vitro. (TIFF) [file pone.0217564.s002.tiff]

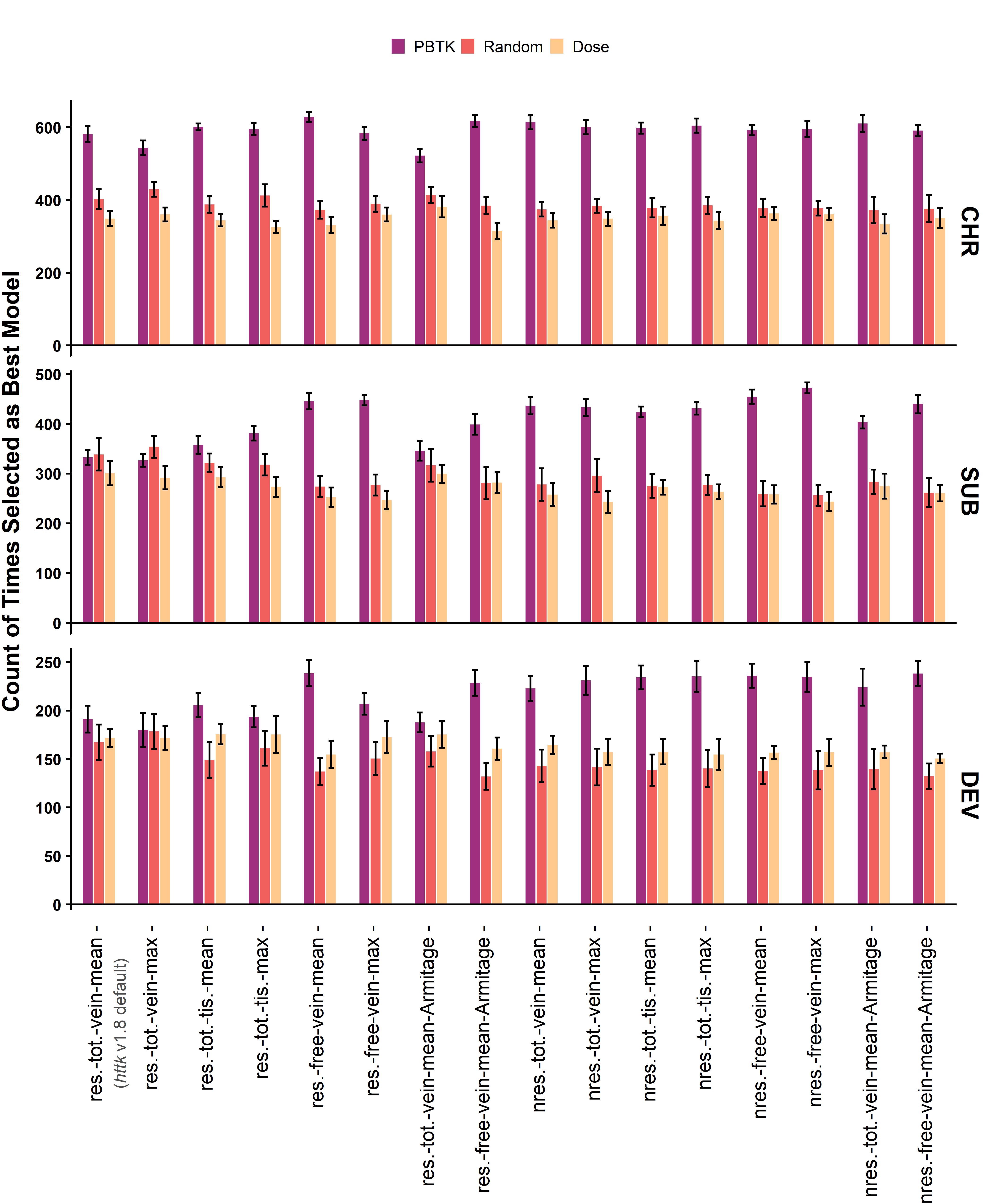

Supplement: S3 Fig — Counts compare in vitro AC50 with predicted in vivo concentration for the endpoint level analysis (top row) and POD level analysis (bottom row) as a function of the assumptions used in application of the PBTK model. Counts are from in vivo-in vitro pairs with at least 5 unique chemicals and are median values from the 10 sets of comparisons. The error bars are plus or minus two standard deviations from the 10 comparisons. Labels on the x-axis indicate assumption set: for clearance (res.–restrictive, nres.–nonrestrictive), concentration selection (tot.–total, free, vein, tis.–tissue, mean, max), and use of the Armitage disposition model to estimate the free concentration in vitro. Results are separated by study type: chronic (CHR), subchronic (SUB), and developmental (DEV). (TIFF) [file pone.0217564.s003.tiff]

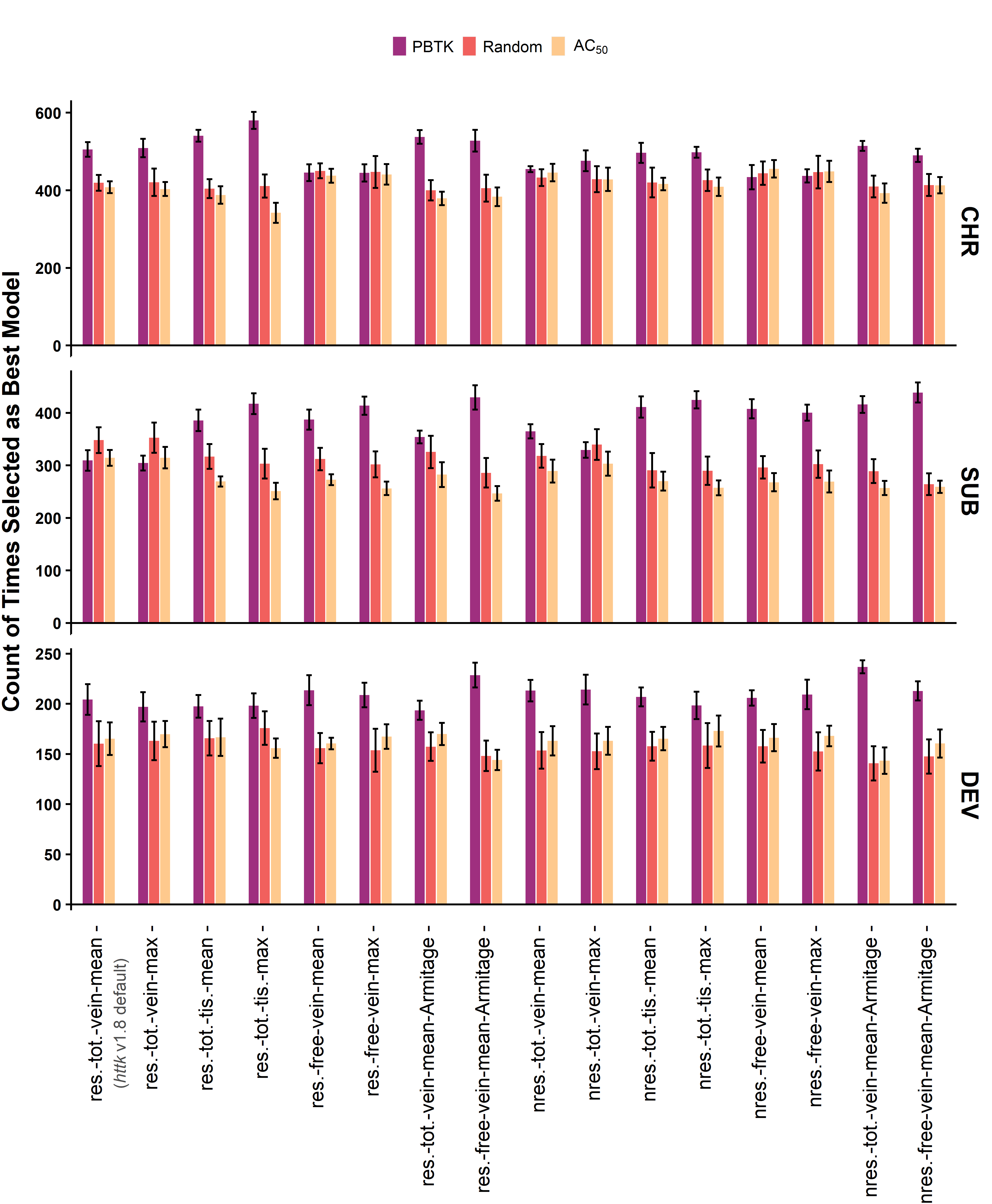

Supplement: S4 Fig — Counts compare in vivo dose with predicted AED from in vitro toxicity assay results for the endpoint level analysis (top row) and POD level analysis (bottom row) as a function of the assumptions used in application of the PBTK model. Counts are from in vivo-in vitro pairs with at least 5 unique chemicals and are median values from the 10 sets of comparisons. The error bars are plus or minus two standard deviations from the 10 comparisons. Labels on the x-axis indicate assumption set: for clearance (res.–restrictive, nres.–nonrestrictive), concentration selection (tot.–total, free, vein, tis.–tissue, mean, max), and use of the Armitage disposition model to estimate the free concentration in vitro. Results are separated by study type: chronic (CHR), subchronic (SUB), and developmental (DEV). (TIFF) [file pone.0217564.s004.tiff]

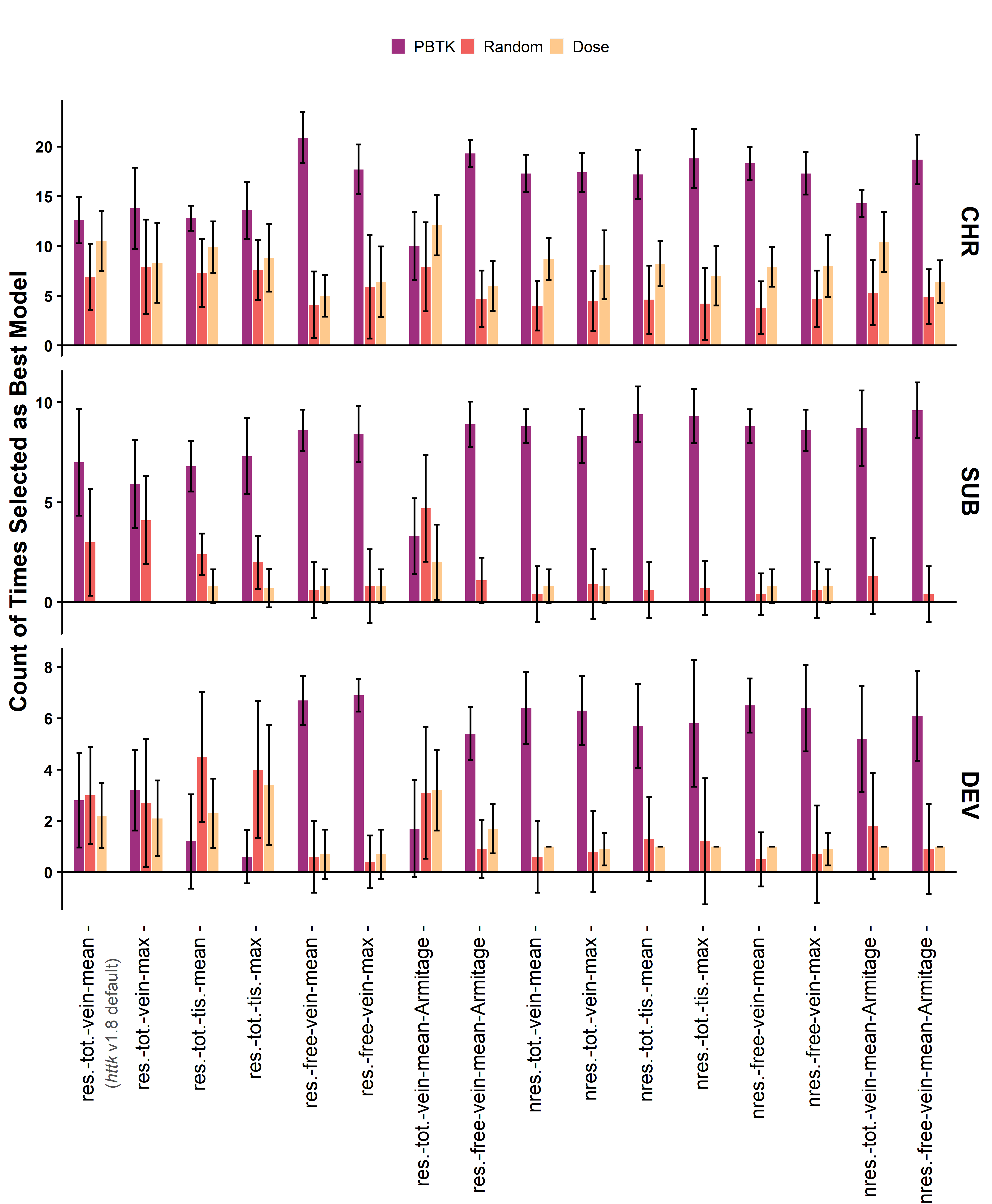

Supplement: S5 Fig — Counts compare in vitro AC50 with predicted in vivo concentration for the endpoint level analysis (top row) and POD level analysis (bottom row) as a function of the assumptions used in application of the PBTK model. Counts are from in vivo-in vitro pairs with at least 20 unique chemicals and are median values from the 10 sets of comparisons. The error bars are plus or minus two standard deviations from the 10 comparisons. Labels on the x-axis indicate assumption set: for clearance (res.–restrictive, nres.–nonrestrictive), concentration selection (tot.–total, free, vein, tis.–tissue, mean, max), and use of the Armitage disposition model to estimate the free concentration in vitro. Results are separated by study type: chronic (CHR), subchronic (SUB), and developmental (DEV). (TIFF) [file pone.0217564.s005.tiff]

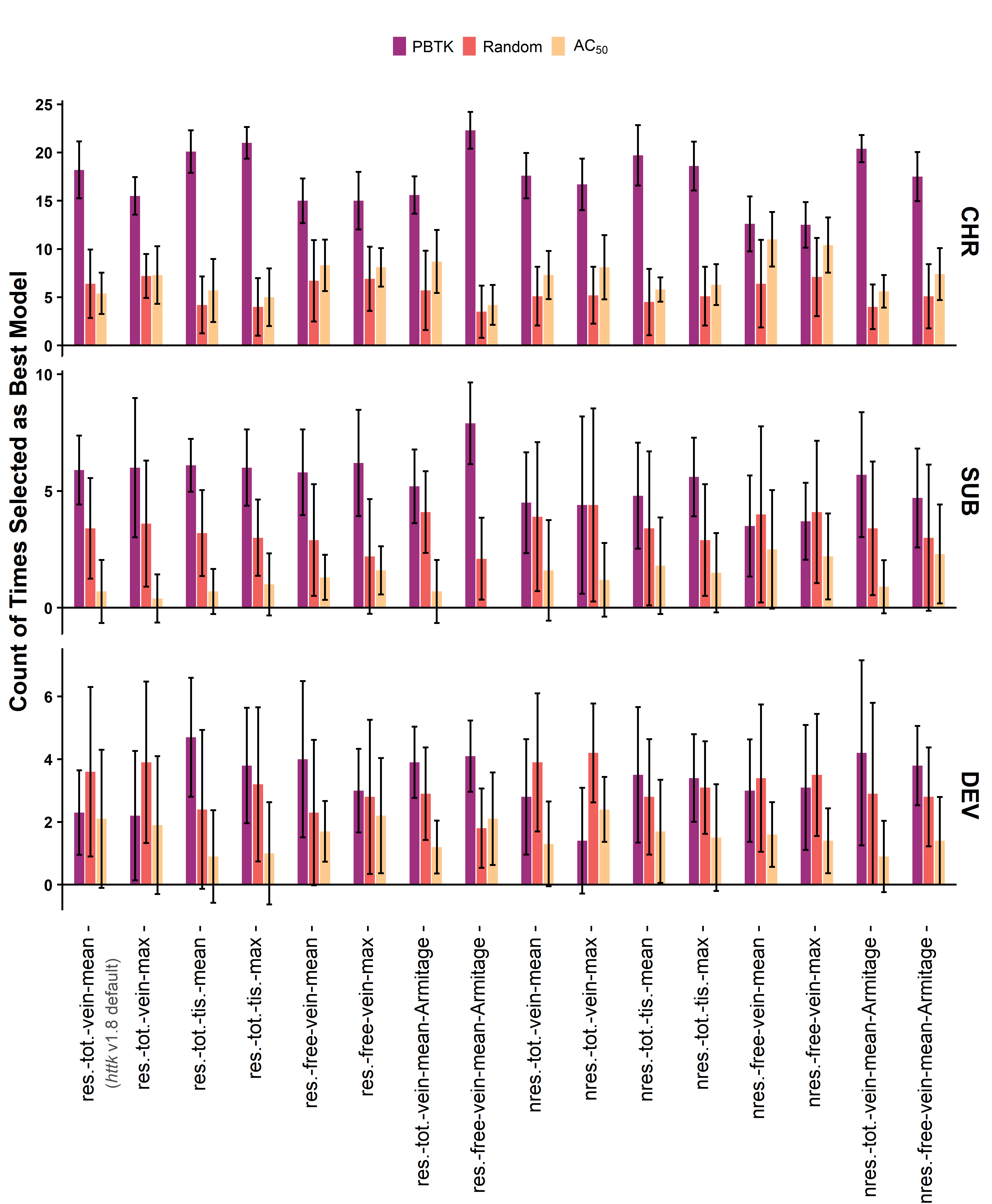

Supplement: S6 Fig — Counts compare in vivo dose with predicted AED from in vitro toxicity assay results for the endpoint level analysis (top row) and POD level analysis (bottom row) as a function of the assumptions used in application of the PBTK model. Counts are from in vivo-in vitro pairs with at least 20 unique chemicals and are median values from the 10 sets of comparisons. The error bars are plus or minus two standard deviations from the 10 comparisons. Labels on the x-axis indicate assumption set: for clearance (res.–restrictive, nres.–nonrestrictive), concentration selection (tot.–total, free, vein, tis.–tissue, mean, max), and use of the Armitage disposition model to estimate the free concentration in vitro. Results are separated by study type: chronic (CHR), subchronic (SUB), and developmental (DEV). (TIFF) [file pone.0217564.s006.tiff]

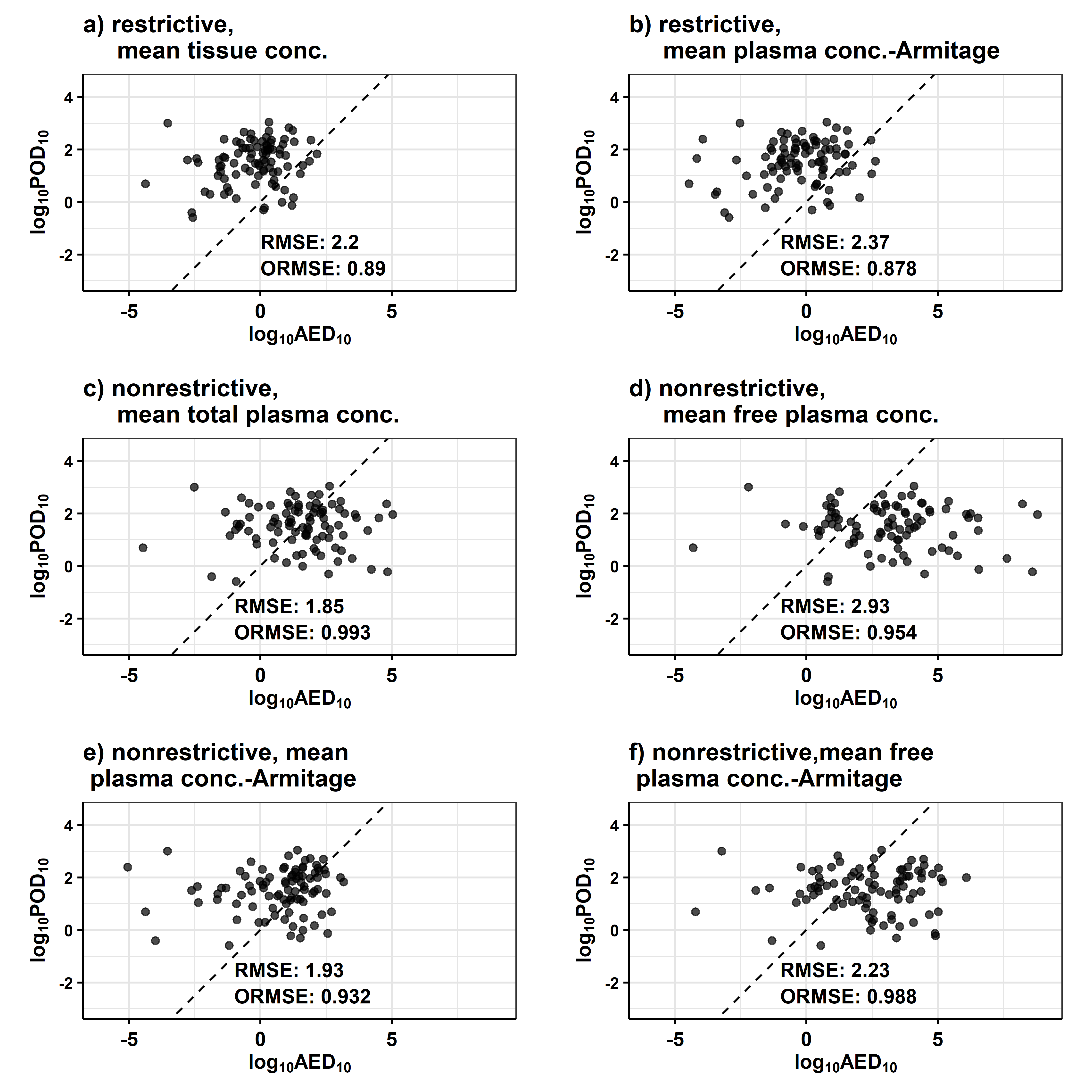

Supplement: S7 Fig — Each point corresponds to a particular chemical. Results are for the assumption set of total mean concentration, and are otherwise indicated by the panel labels: a) restrictive clearance with in vivo mean free total venous plasma concentration, b) restrictive clearance with in vivo mean venous plasma concentration and free concentration in vitro predicted by the Armitage model, c) nonrestrictive clearance with in vivo mean total plasma concentration, d) nonrestrictive clearance with in vivo mean free venous plasma concentration, e) nonrestrictive clearance with in vivo mean total venous plasma concentration and free concentration in vitro predicted by the Armitage model, f) nonrestrictive clearance with in vivo mean free venous plasma concentration and free concentration in vitro predicted by the Armitage model. The dashed lines are y = x lines. Corresponding RMSE and ORMSE (the latter defined for the standardized variables) are also reported. (TIFF) [file pone.0217564.s007.tiff]
