# Supplementary material for: Using the concordance of in vitro and in vivo data to evaluate extrapolation assumptions
Source: PLoS One. 2019 May 28;14(5):e0217564. doi: 10.1371/journal.pone.0217564 (PMC6538186; doi:10.1371/journal.pone.0217564)
Supplement: S2 File — (DOCX) [file pone.0217564.s009.docx]

Abbreviations

AC_50_ – the concentration corresponding to 50% maximal activity in an *in vitro* bioactivity assay, µM

AED – Administered equivalent dose, subscripts may denote the PBTK result or random result (*rand, i*), mg/kg/day

*C* – a vector of concentrations, µM

*Cl_int_* – the intrinsic hepatic clearance, µL/min/million cells

|COR| – the absolute value of the Pearson’s correlation coefficient

DMSO – dimethyl sulfoxide

*f_up_* – the fraction of unbound chemical in plasma

GC-MS – gas chromatography-mass spectrometry

*httk* – the R software package for high-throughput toxicokinetics

HTTK – high-throughput toxicokinetics, includes all associated *in vitro* and *in silico* methods and models

IVIVE – *in vitro* to *in vivo* extrapolation

LC-MS/MS – liquid chromatography tandem mass spectrometry

LOAEL – Lowest observed adverse effect Level

LOEL – Lowest observed effect Level

logD – the log_10_ value of the dissociation constant

logP – the log_10_ value of the octanol-water partition coefficient

MW – molecular weight

ORMSE – the orthogonal root mean square error of the standardized log_10_ transforms of the variables

PBS – phosphate buffered solution

PBTK – physiologically-based toxicokinetics

POD – Point of departure

RED – Rapid Equilibrium Dialysis – the *in vitro* assay used to measure *f_up_*

RMSE – the root mean squared error of a the log_10_ transformed variable

TK – toxicokinetics

Greek symbols

*φ* – a placeholder for a generic standardized variable, Equation 2a

*θ –* a placeholder for a generic standardized variable, Equation 2a

*σ –* the standard deviation

*κ* – the concentration to dose ratio, µM/(mg/kg body mass/day)

Subscripts

*PBTK* – refers to the PBTK result

*rand, i* – refers to the random result, *i* may be 1:10

*10* – after POD or AED, indicates the 10^th^ percentile
