# Supplementary material for: Using the concordance of in vitro and in vivo data to evaluate extrapolation assumptions
Source: PLoS One. 2019 May 28;14(5):e0217564. doi: 10.1371/journal.pone.0217564 (PMC6538186; doi:10.1371/journal.pone.0217564)
Supplement: S3 File — (DOCX) [file pone.0217564.s010.docx]

Assumptions and concentration (i.e, internal dose selections) evaluated in this work

**Clearance**

- restrictive – hepatic clearance depends on *f_up_*
- nonrestrictive – hepatic clearance is independent of *f_up_*

***In vivo* concentration selection**

- mean – use the mean (i.e., average) concentration
- max – use the maximum concentration
- vein or plasma or venous plasma – use the chemical concentration in venous plasma
- tissue – use the concentration in the tissue compartment corresponding to the assay cell type if available, otherwise use the concentration in the rest of body compartment
- total – use the total concentration with respect venous plasma or tissue
- free – use the free venous plasma concentration

***In vitro* concentration selection**

- Armitage or *in vitro* free concentration – use the Armitage *in vitro* disposition model to predict the free concentration in the *in vitro* bioactivity assay
